# Cross-reactive and mono-reactive SARS-CoV-2 CD4+ T cells in prepandemic and COVID-19 convalescent individuals

**DOI:** 10.1371/journal.ppat.1010203

**Published:** 2021-12-29

**Authors:** Alexandra M. Johansson, Uma Malhotra, Yeseul G. Kim, Rebecca Gomez, Maxwell P. Krist, Anna Wald, David M. Koelle, William W. Kwok

**Affiliations:** 1 Benaroya Research Institute at Virginia Mason, Seattle, Washington, United States of America; 2 Virginia Mason Franciscan Health, Seattle, Washington, United States of America; 3 Department of Medicine, University of Washington, Seattle, Washington, United States of America; 4 Vaccine and Infectious Diseases Division, Fred Hutchinson Cancer Research Center, Seattle, Washington, United States of America; 5 Department of Epidemiology, University of Washington, Seattle, Washington, United States of America; 6 Department of Laboratory Medicine and Pathology, University of Washington, Seattle, Washington, United States of America; 7 Department of Global Health, University of Washington, Seattle, Washington, United States of America; University of Rochester Medical Center, UNITED STATES

## Abstract

Class II tetramer reagents for eleven common DR alleles and a DP allele prevalent in the world population were used to identify SARS-CoV-2 CD4+ T cell epitopes. A total of 112, 28 and 42 epitopes specific for Spike, Membrane and Nucleocapsid, respectively, with defined HLA-restriction were identified. Direct *ex vivo* staining of PBMC with tetramer reagents was used to define immunodominant and subdominant T cell epitopes and estimate the frequencies of these T cells in SARS-CoV-2 exposed and naïve individuals. Majority of SARS-CoV-2 epitopes identified have <67% amino acid sequence identity with endemic coronaviruses and are unlikely to elicit high avidity cross-reactive T cell responses. Four SARS-CoV-2 Spike reactive epitopes, including a DPB1*04:01 restricted epitope, with ≥67% amino acid sequence identity to endemic coronavirus were identified. SARS-CoV-2 T cell lines for three of these epitopes elicited cross-reactive T cell responses to endemic cold viruses. An endemic coronavirus Spike T cell line showed cross-reactivity to the fourth SARS-CoV-2 epitope. Three of the Spike cross-reactive epitopes were subdominant epitopes, while the DPB1*04:01 restricted epitope was a dominant epitope. Frequency analyses showed Spike cross-reactive T cells as detected by tetramers were present at relatively low frequency in unexposed people and only contributed a small proportion of the overall Spike-specific CD4+ T cells in COVID-19 convalescent individuals. In total, these results suggested a very limited number of SARS-CoV-2 T cells as detected by tetramers are capable of recognizing ccCoV with relative high avidity and vice versa. The potentially supportive role of these high avidity cross-reactive T cells in protective immunity against SARS-CoV-2 needs further studies.

## Author summary

Previous studies with activation induced marker assays in monitoring antigen-specific CD4+ T cells have shown that common cold coronavirus T cells can cross-react with SARS-CoV-2 antigens and these cross-reactive T cells are present in up to 60% of the unexposed population. In this current study, sets of overlapping peptides for Spike, Membrane, and Nucleocapsid proteins were used to identify epitopes across 11 HLA-DR and 1 HLA-DP alleles in SARS-CoV-2 convalescent samples using peptide-loaded MHC-II tetramers. Using these tetramers *ex vivo*, frequencies of these epitope-specific T cells were estimated in convalescent and pre-pandemic samples. Based on these frequencies, epitopes were stratified into immunodominant and subdominant epitopes. Amino acid sequences of epitopes identified were compared with 4 common cold coronaviruses. Potential cross-reactive epitopes were defined as having ≥67% sequence identity between common cold viruses and SARS-CoV-2. Four potential Spike specific cross-reactive epitopes were identified and functional cross-reactivity was demonstrated. Of the four cross-reactive epitopes identified, three were subdominant epitopes eliciting relatively low frequencies in both unexposed and convalescent subjects. In contrast to the results from the activation induced marker assays, the current data suggests that only a limited number of high avidity SARS-CoV-2 T cells as detected by tetramers are cross-reactive.

## Introduction

Since the first reported index case of Coronavirus disease in December 2019 (COVID-19) [1], severe acute respiratory syndrome coronavirus 2 (SARS-CoV-2) has infected more than 175 million people worldwide in the first 18 months of the pandemic. Though most infected persons have either mild disease or are asymptomatic, approximately 15% of infected persons required hospitalization, with an estimated mortality rate of approximately 0.5–1% worldwide [2]. Epidemiological data have shown that older age, obesity, and other comorbidities, such as diabetes, heart disease, kidney disease, stroke, dementia, and immunosuppression are risk factors for more severe disease [3]. Other data demonstrated that the A blood group gene, certain genetic variants in anti-viral response genes, inflammation related genes, and other unknown genetic variants also contributed to development of severe disease [4–7].

Studies examining T cell responses toward SARS-CoV-2 have been extensive. Several early investigations in this area utilized activation induced marker (AIM) assays, in which cells were stimulated with SARS-CoV-2 pooled peptides overnight. T cells which upregulated activation markers were identified as SARS-CoV-2-specific cells [8–12]. Other investigators used IFN-γ ELISPOT, intracellular cytokine staining, and CFSE dilution-based proliferation assays as readouts [13–18]. CD4+ and CD8+ T cell epitopes for SARS-CoV-2 have also been identified [13,19–21]. More recent studies also used class I tetramer reagents in examining CD8+ T cell responses [12,21–24]. However, *ex vivo* studies of phenotypes and frequencies of CD4+ T cell responses at the epitope level have been very limited. Most of these CD4+ epitopes identified so far do not display an experimentally verified HLA restriction element.

Though SARS-CoV-2 is a newly emerging virus, multiple investigators have reported the presence of SARS-CoV-2-specific T cells in 20%-60% of unexposed persons [8,15,22,25–27]. Since endemic common cold coronaviruses (ccCoV), including NL63 and 229E (alphacoronaviruses) and OC43 and HKU-1 (betacoronaviruses), and SARS-CoV-2 virus are within the same *coronavirinae* subfamily [28,29], pre-existing ccCoV-specific T cells could recognize SARS-CoV-2, and accounts for the presence of SARS-CoV-2 reactive cells in unexposed persons [15,25,26,30,31]. Studies that examined the T cell receptor (TCR) usages of ccCoV and SARS-CoV-2 specific T cells also confirm the presence of these cross-reactive T cells [18,30,32]. These studies raise the possibility that these cross-reactive cells in unexposed persons could potentially mount a more rapid adaptive immune response against the novel SARS-CoV-2 and modulate the clinical outcomes of the disease [33,34]. This scenario was supported by a recent epidemiology study which showed that recent ccCoV infection was associated with less severe COVID-19 [27,35]. However, the extent of this cross-reactivity is unknown and studies that address the frequencies and phenotypes of cross-reactive T cells for a specific epitope in unexposed and COVID-19 persons have not been performed. It also remains unclear whether these cross-reactive T cells play a major role in immune protection. As amino acid sequence similarity for the structural proteins between SARS-CoV-2 and the endemic coronavirus should be less than 35% [36] we reasoned that T cell cross-reactivity between these viruses is minimal.

In the current study, we utilized class II tetramer reagents for epitope identification and examined SARS-CoV-2-specific CD4+ T cells in both COVID-19 convalescent individuals (exposed) and pre-December 2019 SARS-CoV-2 naïve individuals (unexposed). This study included 11 prevalent HLA class II DR alleles and 1 DP allele, covering at least 60% of the world population [37]. Amongst the SARS-CoV-2 T cell epitopes identified in this study, amino acid sequence identity of ≥67% in the core MHC binding region between structural proteins of SARS-CoV-2 and the endemic coronaviruses was used to identify potential cross-reactive T cell epitopes. The 67% cutoff was chosen based on an early study that show 67% amino acid homology was a useful benchmark for consideration of cross-reactivity between class II epitopes [25]. Of the 66 antigenic SARS-CoV-2 Spike peptides identified in this current study, four were predicted to be cross-reactive epitopes between SARS-CoV-2 and ccCoV. Functional cross-reactivity was demonstrated for all four of these SARS-CoV-2 epitopes. Most of the T cell lines that were specific for ccCoV also did not cross recognize SARS-CoV-2. The percentage of Spike-reactive T cells that was cross-reactive in convalescent COVID-19 individuals was also estimated. We show that Spike specific cross-reactive T cells only comprised a very small percentage of the overall Spike specific T cells in COVID-19 convalescent individuals.

## Results

### T cell epitope identification

With PBMCs from COVID-19 convalescent donors (S1 Table), the tetramer guided epitope mapping (TGEM) approach was used to identify CD4+ T cell epitopes within Spike (S), Nucleocapsid (N) and Membrane (M) proteins of SARS-CoV-2 utilizing peptides derived from the US-WA1/2020 strain [38,39]. TGEM inherently includes precise determination of HLA restriction simultaneously with discovery of antigenic peptides. A total of 100 antigenic peptides were identified with HLA restriction, including restriction by DRB1*01:01 (DR0101), DRB1*03:01 (DR0301), DRB1*04:01 (DR0401), DRB1*04:04 (DR0404), DRB1*07:01 (DR0701), DRB1*11:01 (DR1101), DRB1*11:04 (DR1104), DRB1*15:01 (DR1501), DRB3*01:01 (DRB3), DRB4*01:01 (DRB4), DRB5*01:01 (DRB5) and DPB1*04:01 (DP0401) (Table 1). The HLA alleles of the current cohort included 11 of the most common HLA-DR alleles, and the prevalent DP0401 allele. This set of class II alleles covers more than 60% of the world population according to the data from the 18^th^ International HLA and Immunogenetics Workshop [37] (S2 Table). An example of results from the TGEM experiment for a DR0401 person is shown in S1A and S1B Fig. For each class II allele of interest with the exception of DR0404 and DR1104, epitope mapping experiments were carried out in at least two different individuals. Percentage of tetramer positive T cells for a specific epitope for all these TGEM experiments ranged from 0.22% to 32%. The mean percentage of the tetramer positive T cells for each HLA/epitope is listed in Table 1. Higher percentage implied higher frequency of the epitope specific cells under examination. For each allele, epitopes that elicited strong or weak T cell responses were present.

**Table 1:** SARS-CoV-2 Spike peptides identified in tetramer-guided epitope mapping (TGEM). Peptides in red bold are those with ≥67% amino acid identity in the MHC binding region to one of the four ccCoVs. Numbers indicate mean CD4+ Tetramer+ T cells (n = # of positives).

**Spike AA#**	**Sequence**	**DR0101**	**DR0301**	**DR0401**	**DR0404**	**DR0701**	**DR1101**	**DR1104**	**DR1501**	**DRB3**	**DRB4**	**DRB5**	**DP0401**	**Total**
Sp4 25–44	PPAYTNSFTRGVYYPDKVFR	0.585 (2)		3.9 (2)		0.3 (1)				5.06 (2)				4
Sp5 33–52	TRGVYYPDKVFRSSVLHSTQ			12.4 (2)						8.33 (1)				2
Sp6 41–60	KVFRSSVLHSTQDLFLPFFS					0.43 (2)								1
Sp8 57–76	PFFSNVTWFHAIHVSGTNGT	1.78 (1)				0.82 (1)			4.23 (4)					3
Sp13 97–116	KSNIIRGWIFGTTLDSKTQS								1.52 (3)					1
Sp17 129–148	KVCEFQFCNDPFLGVYYHKN												0.63 (2)	1
Sp21 161–180	SSANNCTFEYVSQPFLMDLE												25.7 (2)	1
Sp24 185–204	NFKNLREFVFKNIDGYFKIY											2.26 (4)		1
Sp25 193–212	VFKNIDGYFKIYSKHTPINL											2.65 (4)		1
Sp26 201–220	FKIYSKHTPINLVRDLPQGF					0.99 (2)				2.84 (2)				2
Sp27 209–228	PINLVRDLPQGFSALEPLVD									2.62 (2)				1
Sp29 217–244	PLVDLPIGINITRFQTLLAL				0.72 (1)									1
Sp30 233–252	INITRFQTLLALHRSYLTPG				0.97 (1)				0.23 (2)			0.63 (4)		3
Sp38 297–316	SETKCTLKSFTVEKGIYQTS			4.82 (2)										1
Sp39 305–324	SFTVEKGIYQTSNFRVQPTE			0.79 (2)		4.14 (2)								2
Sp40 313–332	YQTSNFRVQPTESIVRFPNI	1.85 (3)		8.8 (2)										2
Sp41 321–340	QPTESIVRFPNITNLCPFGE				0.49 (1)				4.6 (2)					2
Sp43 337–356	PFGEVFNATRFASVYAWNRK											7.06 (4)	0.34 (2)	2
Sp44 345–364	TRFASVYAWNRKRISNCVAD		4.93 (1)				3.61 (2)					7.05 (4)		3
Sp45 353–372	WNRKRISNCVADYSVLYNSA		1.78 (2)								3.99 (1)			2
Sp50 393–412	TNVYADSFVIRGDEVRQIAP		0.34 (2)							5.57 (2)				2
Sp51 401–420	VIRGDEVRQIAPGQTGKIAD									0.74 (1)				1
Sp54 425–444	LPDDFTGCVIAWNSNNLDSK								1.37 (3)					1
Sp55 433–452	VIAWNSNNLDSKVGGNYNYL								2.05 (3)					1
Sp56 441–460	LDSKVGGNYNYLYRLFRKSN						2.72 (2)	0.53 (1)						2
Sp57 449–468	YNYLYRLFRKSNLKPFERDI						1.02 (1)	0.63 (1)		0.29 (2)				3
Sp58 457–476	RKSNLKPFERDISTEIYQAG			17.25 (2)						5.58 (2)				2
Sp61 481–500	NGVEGFNCYFPLQSYGFQPT											1.11 (3)		1
Sp62 489–508	YFPLQSYGFQPTNGVGYQPY											0.72 (3)		1
Sp64 505–524	YQPYRVVVLSFELLHAPATV	14.73 (3)												1
Sp65 513–532	LSFELLHAPATVCGPKKSTN	11.2 (3)												1
Sp68 537–556	KCVNFNFNGLTGTGVLTESN	1.9 (3)												1
Sp70 553–572	TESNKKFLPFQQFGRDIADT											2.37 (3)		1
Sp79 625–644	HADQLTPTWRVYSTGSNVFQ					2.03 (2)								1
Sp80 633–652	WRVYSTGSNVFQTRAGCLIG					0.48 (2)								1
Sp85 673–692	SYQTQTNSPRRARSVASQSI					1.27 (1)								1
Sp86 681–700	PRRARSVASQSIIAYTMSLG					2.18 (2)								1
Sp87 689–708	SQSIIAYTMSLGAENSVAYS			1.45 (2)	1.8 (1)	2.55 (2)			2.53 (4)					4
Sp90 713–732	AIPTNFTISVTTEILPVSMT					6.16 (2)								1
Sp94 745–764	DSTECSNLLLQYGSFCTQLN								5.64 (3)					1
Sp95 753–772	LLQYGSFCTQLNRALTGIAV								1.93 (2)					1
Sp98 777–796	NTQEVFAQVKQIYKTPPIKD							0.81 (1)				2.58 (4)		2
Sp101 801–820	NFSQILPDPSKPSKRSFIED		6.6 (2)	22.7 (2)	0.89 (1)									3
Sp102 809–828	PSKPSKRSFIEDLLFNKVTL												1.58 (2)	1
Sp103 817–836	FIEDLLFNKVTLADAGFIKQ												1.92 (1)	1
Sp108 857–876	GLTVLPPLLTDEMIAQYTSA		1.64 (2)											1
Sp109 865–884	LTDEMIAQYTSALLAGTITS								10.63 (4)					1
Sp112 889–908	GAGAALQIPFAMQMAYRFNG	2.22 (3)				4 (2)		1.52 (1)				2.02 (4)		4
Sp113 897–916	PFAMQMAYRFNGIGVTQNVL	1.68 (3)					0.55 (2)	3.06 (1)				2.14 (4)		4
Sp114 905–924	RFNGIGVTQNVLYENQKLIA							12.4 (1)	1.5 (1)					2
Sp115 913–932	QNVLYENQKLIANQFNSAIG						0.95 (2)	16.7 (1)	1.62 (1)					3
Sp117 929–948	SAIGKIQDSLSSTASALGKL			4.07 (2)	0.33 (1)									2
Sp118 937–956	SLSSTASALGKLQDVVNQNA			2.34 (2)										1
Sp120 953–972	NQNAQALNTLVKQLSSNFGA	0.95 (2)			3.17 (1)		0.67 (2)	15.9 (1)						4
Sp121 961–980	TLVKQLSSNFGAISSVLNDI	0.68 (3)			1.89 (1)									2
Sp124 985–1004	DKVEAEVQIDRLITGRLQSL		1.18 (2)											1
Sp125 993–1012	IDRLITGRLQSLQTYVTQQL	0.86 (3)			0.5 (1)									2
Sp126 1001–1020	LQSLQTYVTQQLIRAAEIRA								3.52 (4)					1
Sp127 1009–1028	TQQLIRAAEIRASANLAATK			3.79 (2)	1.98 (1)									2
Sp128 1017–1036	EIRASANLAATKMSECVLGQ			0.22 (2)	1.37 (1)									2
Sp133 1057–1076	PHGVVFLHVTYVPAQEKNFT											0.67 (4)		1
Sp135 1073–1092	KNFTTAPAICHDGKAHFPRE		0.75 (1)											1
Sp138 1097–1116	SNGTHWFVTQRNFYEPQIIT			3.84 (2)										1
Sp139 1105–1124	TQRNFYEPQIITTDNTFVSG		1.6 (2)	4.68 (2)										2
Sp140 1113–1132	QIITTDNTFVSGNCDVVIGI		0.5 (2)	2.18 (2)										2
Sp157 1249–1268	SCGSCCKFDEDDSEPVLKGV									3.32 (2)				1
	**Total Spike (n):**	11	9	15	11	12	6	8	13	9	1	12	5	112
**Membrane AA#**	**Sequence**	**DR0101**	**DR0301**	**DR0401**	**DR0404**	**DR0701**	**DR1101**	**DR1104**	**DR1501**	**DRB3**	**DRB4**	**DRB5**	**DP0401**	
Mp2 9–28	TVEELKKLLEQWNLVIGFLF					2.26 (1)								1
Mp6 36–52	QFAYANRNRFLYIIKLI						1.49 (1)							1
Mp9 65–84	FVLAAVYRINWITGGIAIAM	7.17 (2)												1
Mp12 89–180	GLMWLSYFIASFRLFARTRS	1.67 (1)					0.93 (1)							2
Mp13 97–116	IASFRLFARTRSMWSFNPET			2.61 (2)		1.63 (1)	1.1 (1)							3
Mp14 105–124	RTRSMWSFNPETNILLNVPL	3.28 (2)		0.48 (1)										2
Mp19 145–164	LRGHLRIAGHHLGRCDIKDL		0.76 (1)				16.2 (1)	7.66 (1)			0.99 (1)			4
Mp20 153–172	GHHLGRCDIKDLPKEITVAT						21.3 (1)	2.44 (1)						2
Mp21 161–180	IKDLPKEITVATSRTLSYYK		2.38 (2)						1.14 (1)					2
Mp22 169–188	TVATSRTLSYYKLGASQRVA		2.41 (2)	3.17 (1)		8.67 (2)	0.26 (1)					6.04 (3)		5
Mp23 177–196	SYYKLGASQRVAGDSGFAAY			2.8 (2)		9.29 (2)	0.22 (1)					6.25 (3)		4
Mp26 201–220	IGNYKLNTDHSSSSDNIALL			5.47 (2)										1
	**Total Membrane (n):**	3	3	5	ND	4	7	2	1	0	1	2	ND	28
**Nucleocapsid AA#**	**Sequence**	**DR0101**	**DR0301**	**DR0401**	**DR0404**	**DR0701**	**DR1101**	**DR1104**	**DR1501**	**DRB3**	**DRB4**	**DRB5**	**DP0401**	
Np6 41–60	RPQGLPNNTASWFTALTQHG			1.9 (1)										1
Np7 49–68	TASWFTALTQHGKEDLKFPR			2.49 (2)								21.24 (4)		2
Np11 81–100	DDQIGYYRRATRRIRGGDGK						5.85 (2)					2.06 (4)		2
Np12 89–108	RATRRIRGGDGKMKDLSPRW											0.74 (4)		1
Np16 121–140	LPYGANKDGIIWVATEGALN			4.59 (2)	6.47 (1)	1.08 (2)								3
Np17 129–148	GIIWVATEGALNTPKDHIGT			3.96 (2)	4.10 (1)	0.86 (2)								3
Np21 161–180	LPQGTTLPKGFYAEGSRGGS			1.59 (2)										1
Np22 169–188	KGFYAEGSRGGSQASSRSSS			2.08 (2)										1
Np28 217–236	AALALLLLDRLNQLESKMSG		15.22 (2)				31.9 (1)	26.1 (1)		8.36 (2)				4
Np33 257–276	KPRQKRTATKAYNVTQAFGR					2.77 (2)						10.12 (4)		2
Np34 265–284	TKAYNVTQAFGRRGPEQTQG					3.03 (2)						10.12 (4)		2
Np36 281–300	QTQGNFGDQELIRQGTDYKH										3.44 (1)			1
Np37 289–308	QELIRQGTDYKHWPQIAQFA										5.11 (1)			1
Np38 297–316	DYKHWPQIAQFAPSASAFFG					1.32 (2)								1
Np39 305–324	AQFAPSASAFFGMSRIGMEV					1.30 (2)	3.29 (1)							2
Np40 313–332	AFFGMSRIGMEVTPSGTWLT			3.94 (2)	1.73 (1)		2.03 (2)							3
Np41 321–340	GMEVTPSGTWLTYTGAIKLD					6.37 (2)						1.39 (3)		2
Np42 329–348	TWLTYTGAIKLDDKDPNFKD		19.5 (2)			5.12 (2)						1.51 (3)		3
Np44 345–364	NFKDQVILLNKHIDAYKTFP						3.23 (2)	14.9 (1)					0.16 (1)	3
Np45 353–372	LNKHIDAYKTFPPTEPKKDK						1.07 (1)						0.51 (1)	2
Np49 385–404	RQKKQQTVTLLPAADLDDFS	1.5 (3)												1
Np51 401–419	DDFSKQLQQSMSSADSTQA						1.32 (1)							1
**Spike AA#**	**Total Nucleocapsid (n):**	1	2	7	3	8	7	2	0	1	2	7	2	42

### *Ex vivo* staining of PBMC from COVID-19 convalescent and SARS-CoV-2 unexposed persons

Specific tetramer reagents for epitopes identified in TGEM were used to examine the frequency of SARS-CoV-2 T cells in PBMC of exposed and unexposed persons (S1 Table). As multiple epitopes within three viral antigens were examined per sample, some epitopes were pooled together for analysis. A combinatorial tetramer staining approach that included two subsequent enrichment cycles of different tetramer labeled cells was used for these experiments [40]. With this approach, SARS-CoV-2-specific T cells with up to 22 different epitope specificities and additional control epitopes were analyzed within a single sample of 10–20 million cryopreserved PBMC. A schematic depiction of this approach is shown in S2A Fig. S3 Table shows the different tetramer panels used. Control tetramers, such as influenza-specific tetramers, were also included. For some experiments, SARS-CoV-2 tetramer reagents for alleles within the same haplotype, i.e. DR0301 together with DRB3, and DR1501 with DRB5, were used to stain specific PBMC samples to provide an integrated understanding of the overall T cell response in limited PBMC specimens.

**Fig 1:**
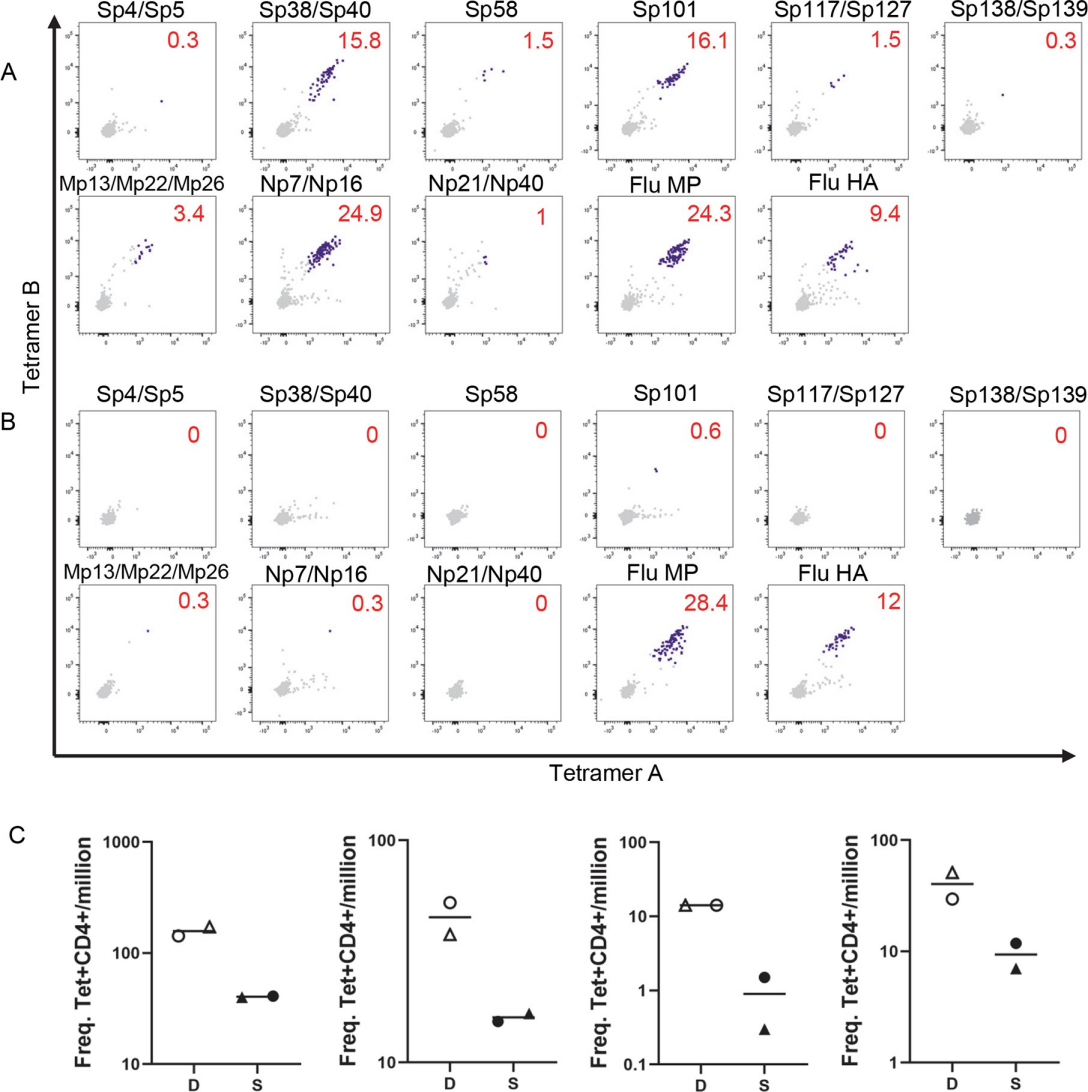
Analysis of SARS-CoV-2-reactive CD4+ T cells by direct *ex vivo* staining of PBMC with class II tetramer reagents. (A-B) PBMCs were incubated with two rounds of a panel of combinatorial peptide-specific tetramers and tetramer positive cells were enriched through a magnetic column before analysis. Sp4 (S_25-44_), Sp5 (S_33-52_), Sp38 (S_297-316_), Sp39 (S_305-324_), Sp58 (S_457-476_), Sp101 (S_801-820_), Sp117 (S_929-948_), Sp127 (S_1009-1028_), Sp138 (S_1097-1116_), Sp139 S_1105-1124_), Mp13 (M_97-116_), Mp22 (M_169-188_), Mp26 (M_201-220_), Np7 (N_49-68_), Np16 (N_121-140_), Np21 (N_161-180_), Np40 (N_313-332_), Flu MP (Flu MP_61-75_ and MP_97-116_), Flu HA (Flu HA_269-283_ and HA_306-318_). Tetramers A and B are tetramers with identical epitope as indicated in each panel, but with different fluorochromes. (A) Representative example of DR0401 SARS-CoV-2 exposed individual. Numbers indicate the frequency of tetramer-positive cells per million CD4+ T cells. (B) Representative example of DR0401 SARS-CoV-2 unexposed individual. Numbers indicate the frequency of tetramer-positive cells per million CD4+ T cells. (C) Frequency of T cells specific for dominant (D) and subdominant (S) epitopes for DR0401 SARS-CoV-2 exposed individuals. Each graph represents an exposed individual. Sp38/Sp40 (open circle), Sp101 (open triangle), Sp117/Sp127 (closed circle), and Sp138/Sp139 (closed triangle).

Examples of a typical staining of a DR0401 exposed person and an unexposed person are shown in Fig 1A and 1B, respectively. For the DR0401 exposed person, S_297-216_, S_313-332_, and S_801-820_ epitope-specific T cells were detected at higher frequencies compared to S_929-948_, S_1009-1028_, S_1097-1116,_ and S_1105-1124_ epitope-specific T cells. A similar pattern of T cell epitope hierarchy was observed in three other DR0401 COVID-19-convalescent individuals studied (Fig 1C).

**Fig 2:**
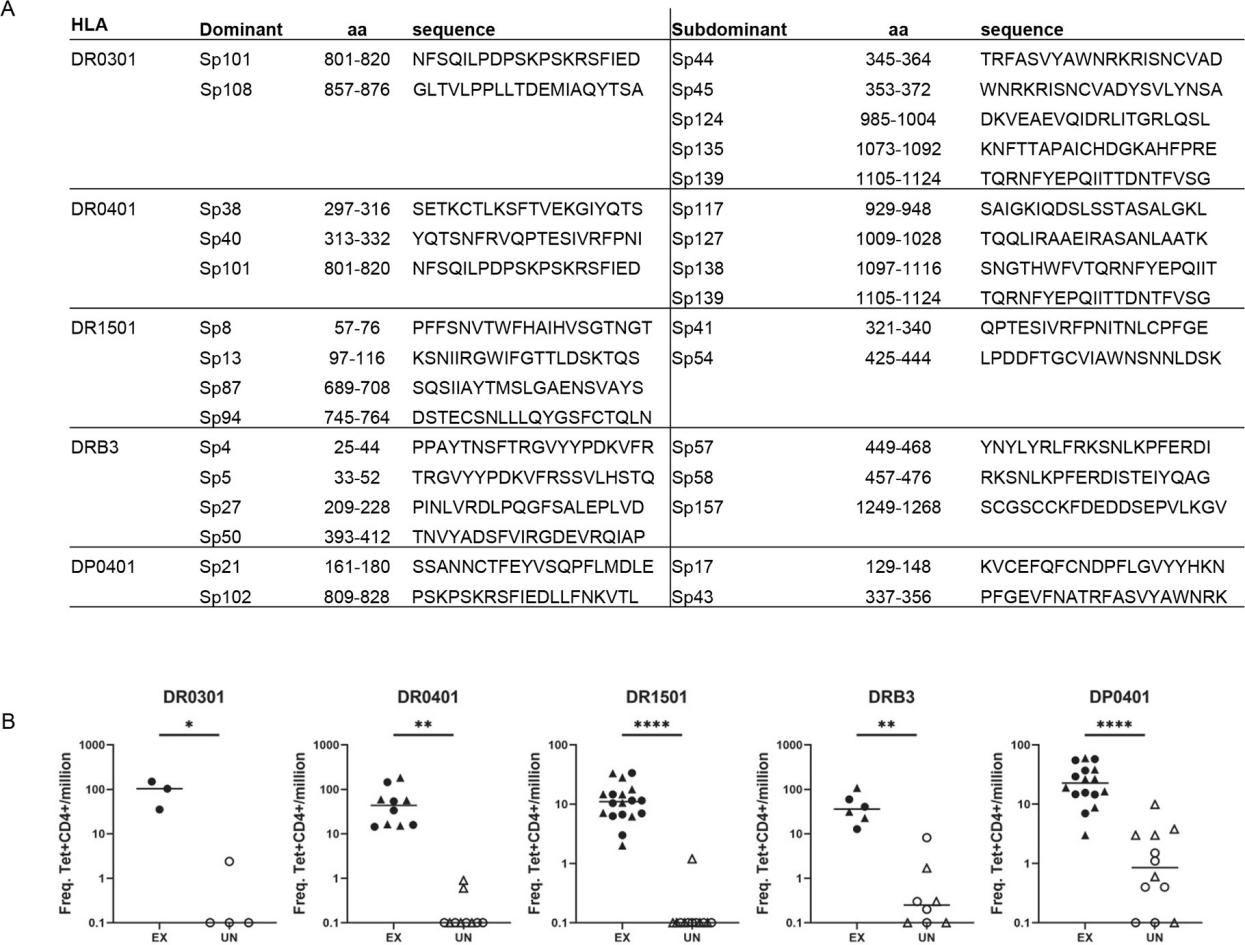
Dominant and subdominant epitopes. (A) Table of dominant and subdominant epitopes for indicated HLAs used in this study. (B) Frequency of T cells specific for dominant epitopes of SARS-CoV-2 exposed (Ex) and unexposed (UN) individuals for each HLA. DR0301 Sp101/Sp108 (circle); DR0401 Sp38/Sp40 (circle), Sp101 (triangle); DR1501 Sp8/Sp13 (circle), Sp87/Sp94 (triangle); DRB3 Sp4/Sp5 (circle), Sp27/Sp50 (Triangle); DP0401 Sp21 (circle), Sp102 (triangle). Student’s unpaired t-test; * p≤0.05, ** p≤0.01, *** p≤0.001, **** p≤0.0001.

For a particular HLA, if the epitope-specific T cells were consistently present at higher frequencies compared to other epitopes of that specific antigen for multiple individuals, these epitopes were designated as immunodominant epitopes. Epitopes that consistently elicited low T cell responses were designated as subdominant epitopes. Dominant and subdominant Spike epitopes for DR0301, DRB3, DR1501 and DP0401 were also identified, and these epitopes are listed in Figs 2A and S3. The frequencies of T cells for these dominant epitopes were significantly higher in exposed persons compared to unexposed persons (Fig 2B).

**Fig 3:**
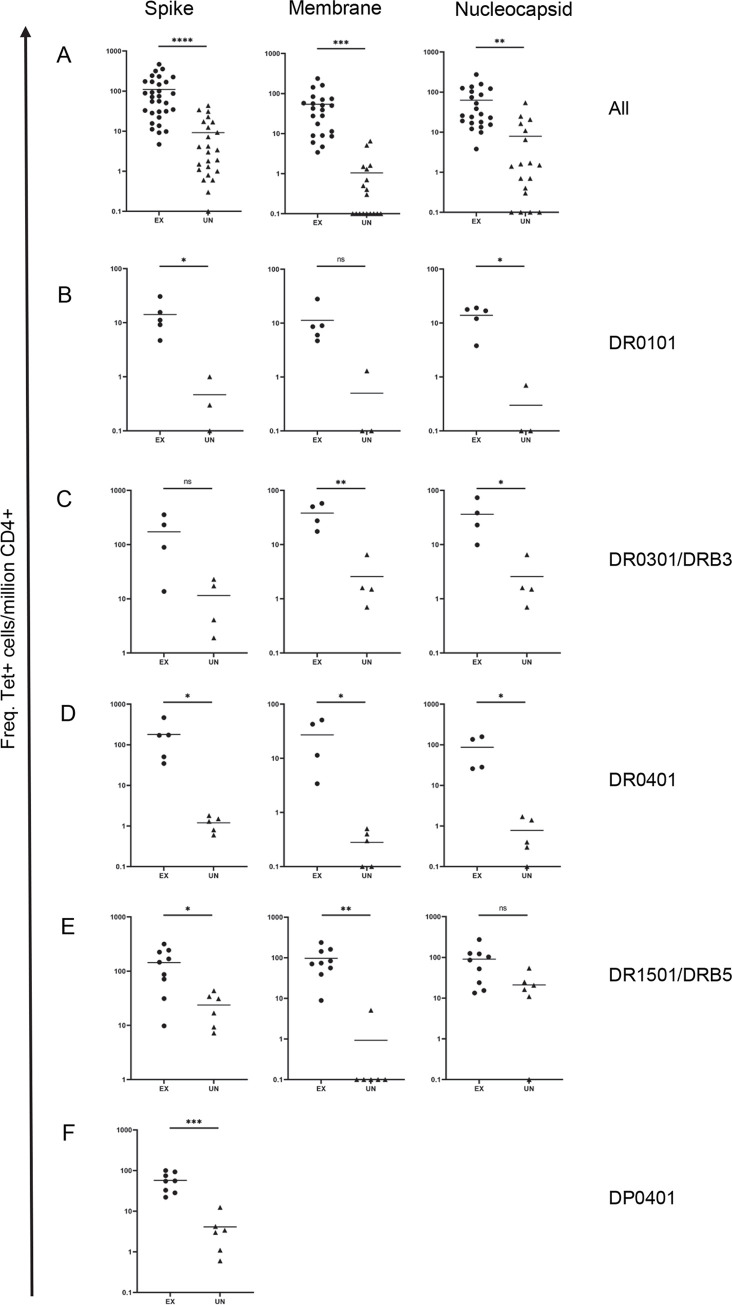
Summary of total frequencies of SARS-CoV-2-reactive CD4+ T cells from *ex vivo* tetramer staining of PBMC from SARS-CoV-2 exposed and unexposed individuals. (A) All HLA frequencies. (n_S_ = 31 exposed, 22 unexposed; n_M_ = 22 exposed, 18 unexposed; n_N_ = 22 exposed, 18 unexposed). (B) DR0101-specific frequencies. (n = 5 exposed, 3 unexposed). (C) DR0301/DRB3-specific frequencies. (n = 4 exposed, 4 unexposed). (D) DR0401-specific frequencies. (n_S_ = 5 exposed, 5 unexposed; n_M_ = 4 exposed, 5 unexposed; n_N_ = 4 exposed, 5 unexposed). (E) DR1501/DRB5-specific frequencies. (n = 9 exposed, 6 unexposed). (F) DP0401-specific frequencies. (n = 8 exposed, 6 unexposed). Student’s unpaired t-test; * p≤0.05, ** p≤0.01, *** p≤0.001, **** p≤0.0001.

Frequencies of DR- or DP-epitope-specific cells for each viral protein restricted by a specific class II allele examined were summed to determine the total frequencies of S, N, and M protein-specific CD4+ cells restricted by a specific allele in PBMC for each person (Fig 3A–3F). For a cohort with five DR0101, four DR0301-DRB3, five DR0401, nine DR1501-DRB5, and eight DP0401 exposed persons, and a total of 22 samples from HLA-matched unexposed individuals, frequencies of S, N, and M reactive T cells were higher in exposed persons compared to unexposed (Fig 3A). When this cohort was stratified by HLA, exposed persons had significantly higher frequencies of SARS-CoV-2 specific T cells compared to unexposed persons with identical HLA for all proteins examined, with the exception of responses to S for DR0301 individuals, to M for DR0101 individuals, and to N for DR1501 individuals.

### SARS-COV-2 Spike T cells and ccCoV Spike T cells cross reactivity

For identification of cross-reactive epitopes, we searched for amino acid homology between the identified SARS-CoV-2 S epitopes and S proteins of SARS-CoV-1 and four ccCoV, NL63, 229E, OC43 and HKU-1. As the SARS-CoV-2 epitopes identified were 20 amino acids (AA) in length, the netMHCIIpan 4.0 prediction tool was used to identify the 9 AA core that constituted the minimum T cell epitope region within the 20mers [41]. The multiple sequence alignment program MUSCLE was used to align the amino acids for the S protein of the 6 viruses. Amongst the 66 SARS-CoV-2 S antigenic peptides identified, only five peptides had more than 6 (≥67%) AA sequence identity to ccCoV in the 9 AA core region (Tables 1 and 2). None of these SARS-CoV-2 core T cell epitope regions had AA sequences completely identical to ccCoV. As S_809-828_ and S_817-836_ contained an identical DP-restricted T cell epitope IEDLLFNKV within S_818-826_, a total of four S reactive T cell epitopes that could potentially elicit cross-reactive T cells were identified.

**Table 2:** FASTA alignment of potential SARS-CoV-2/ccCoV Spike cross-reactive epitopes. Black bold indicates the putative MHC-II binding motif as predicted by NetMHCII pan 4.0. Red indicates an amino acid different from SARS-CoV-2.

HLA/Epitope	Amino Acid Sequence
**DR0101/DR0401**	
SARS-CoV-2_961-980_	tlvkqlssn**FGAISSVLN**di----
NL63_1034-1050_	-------hn**F**Q**AIS**NSIQaiydrl
229E_849-865_	---sqlrqn**F**Q**AISS**SIQai----
OC43_1058-1074_	-----lsnr**FGAIS**AS**L**Qeils--
HKU-1_1049-1068_	---qqlfnk**FGAISS**S**L**Qeilsr-
**DR0301**	
SARS-CoV-2_985-1004_	dkveae**VQIDRLITG**rlqsl---
NL63_1052-1068_	-siqadQ**Q**V**DRLITG**rla-----
229E_872-888_	--iqadQ**Q**V**DRLITG**rlaa----
OC43_1081-1097_	----aeA**QIDRLI**N**G**rltaln--
HKU-1_1073-1092_	---eaq**VQIDRLI**N**G**rltalnay
**DR1501**	
SARS CoV-2_1001-1020_	-----lqs**LQTYVTQQL**iraaeira
NL63_1062-1078_	litgrlaa**L**NAF**V**S**Q**V**L**--------
229E_884-900_	---grlaa**L**NVF**V**SHT**L**tky-----
OC43_1093-1109_	-----lta**L**NA**YV**S**QQL**sdstl---
HKU-1_1081-1100_	lingrlta**L**NA**YV**S**QQL**sdi-----
**DP0401**	
SARS-CoV-2_809-828_	pskpskrsf**IEDLLFNKV**tl-------
NL63_867-883_	---iagrsaL**EDLLF**S**KV**vt-------
229E_690-706_	-------sa**IED**I**LF**S**K**Ltsgl-g---
OC43_911-927_	------rsa**IEDLLF**D**KV**klsdv----
HKU-1_905-924_	-------sfF**EDLLF**D**KV**klsdvgfve

In addition to the potential cross-reactive S epitopes, AA sequence comparisons of M and N between SARS-CoV-2 and ccCoV show that SARS-CoV-2 M_97-116_, M_105-124_, and N_121-140_ also have ≥67% AA sequence identity (Table 1). Overall, of the 100 antigenic peptides identified in this study, 8 (8%) have ≥67% AA sequence identity, suggesting the possibility of T cell cross-reactivity for these epitopes.

**Fig 4:**
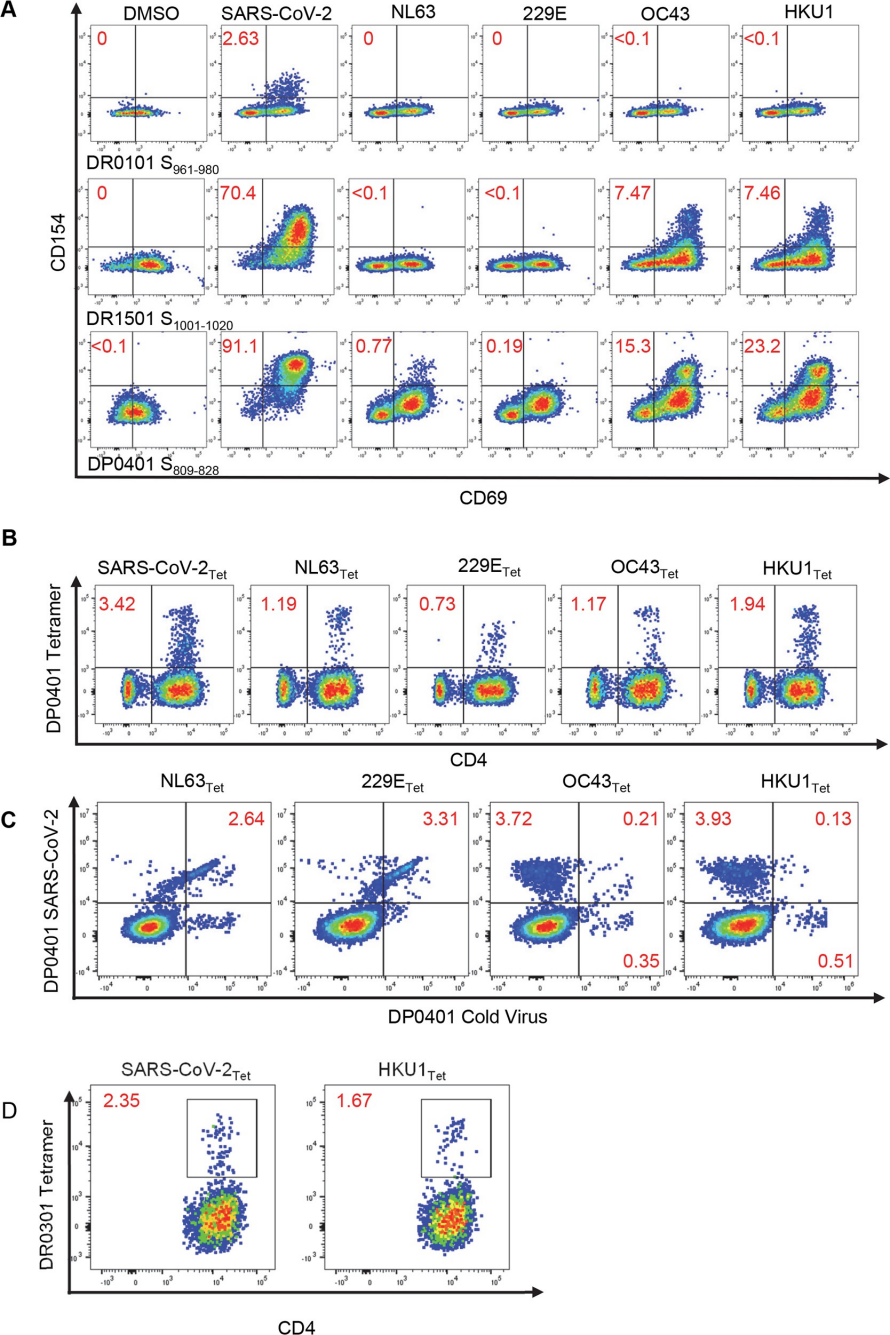
SARS-CoV-2 Cross-reactive T cells. (A) CD154 upregulation assay of potential cross-reactive epitopes. Top panel: DR0101 SARS- CoV-2 S_961-980_, middle panel: DR1501 SARS-CoV-2 S_1001-1020_, and bottom panel: DP0401 SARS-CoV-2 S_809-828_. SARS-CoV-2 peptide-specific cell lines were generated and activated with peptides for five hours from SARS-CoV2 or its equivalent in ccCoV (as listed in Table 2). Cells were then stained with anti-CD154 and anti-CD69. Numbers indicate percentage of CD154+CD69+ T cells. DMSO was used as negative control. (B) PBMC from a DP0401 convalescent individual were stimulated SARS-CoV-2 S_809-828_ peptide and cultured for 14 days. Cells were stained with tetramers containing SARS-CoV-2 S_809-828_ or equivalent ccCoV peptides. Numbers indicate the percentage of CD4+ tetramer+ T cells. (C) PBMC from a DP0401 unexposed individual were stimulated with SARS-CoV-2 S_809-828_ peptide and cultured for 14 days. Cells were stained with SARS-CoV-2 S_809-828_ tetramers or equivalent ccCoV tetramers. Numbers indicate percentage of CD4+ T cells. (D) PBMC from a DR0301 vaccinated individual were stimulated with SARS-CoV-2 S_985-1004_ peptide and cultured for 14 days. Cells were stained with SARS-CoV-2 S_809-828_ or equivalent HKU-1 tetramer. Numbers indicated percentage of CD4+ T cells.

For confirmation of whether AA sequence homology at the sequence level can be translated to T cell cross-reactivity, potentially cross-reactive SARS-CoV-2 T cell lines that recognize the DR0101-, DR1501- and DP0401-restricted cross-reactive Spike epitopes to ccCoV were generated by sorting SARS-CoV-2 tetramer positive cells followed by expansion from exposed persons for functional studies. A CD154 upregulation assay was used to evaluate the ability of these three different SARS-CoV-2 S-specific T cell lines to recognize the corresponding regions of ccCoV S epitopes. The CD154 upregulation assay, a potentially more sensitive approach in detecting low avidity interactions, was performed for these cross-reactivity experiments. Though the DR0101 S_961-980_ lines could not recognize any ccCoV sequences tested, the DR1501 SARS-CoV-2 S_1001-1020_ cell line was partially activated relatively by the OC43 S_1093-1109_ and HKU-1 S_1081-1100_ peptides. The DP0401 SARS-CoV-2 S_809-828_ line was activated by NL63 S_867-883_, OC43 S_911-927_ and HKU-1 S_905-924_ peptides, but not the homologous 229E S_690-706_ peptide (Fig 4A). The cross-reactivity of DP0401 restricted SARS-CoV-2 S_809-828_ T cells was also confirmed by positive staining of another DP0401 SARS-CoV-2 S_809-828_ line obtained from another DP0401 COVID-19 convalescent person with DP0401 NL63 S_867-883_, DP0401 OC43 S_911-927_, DP0401 229E S_690-706_ and DP0401 HKU-1 S_1081-1100_ tetramers (Fig 4B). Slightly different results were observed in a DP0401 SARS-CoV-2 S_809-828_ line from a third unexposed person. For this particular SARS-CoV-2 S_809-828_ line, both NL63 S_867-883_ and 229E S_690-706_ tetramers gave strong signals, but less than 6% of cells in this line recognized the OC43 S_911-927_ and HKU-1 S_905-924_ tetramers (Fig 4C). In addition to the DR1501 and DP0401 restricted T cell cross-reactivity described above, we were able to demonstrate that the DR0301 HKU-1 S_1073-1092_ tetramers could be used to stain a DR0301 SARS-CoV-2 S_985-1004_ T cell line, indicating the cross-reactive nature of this DR0301 restricted T cell line (Fig 4D).

For the cross-reactivity experiments with SARS-CoV-2 T cell lines generated by *in vitro* stimulation with SARS-CoV-2 peptides (Fig 4), the cognate SARS-CoV-2 peptides usually elicited a stronger signal by either tetramer staining or CD154 upregulation assays compared to those elicited by the ccCoV-2 peptides.

For further confirmation of cross-reactivity and to detect the presence of cross-reactive T cells in unexposed persons, DR1501 OC43 S_1093-1109_ and DP0401-restricted NL63 S_867-883_ or HKU-1 S_905-924_ reactive cell lines were isolated from SARS-CoV-2-unexposed persons by sorting of ccCoV S tetramer positive cells. CD154 upregulation assays were performed to show that DR1501-restricted T cell lines elicited from ccCoV OC43 S_1093-1109_ peptide stimulation (S4A Fig) and DP0401 restricted T cell line elicited from either NL63 S_867-883_ or HKU-1 S_905-924_ peptide stimulation (S4B and S4C Fig) were capable of recognizing the corresponding regions of SARS-CoV-2 protein and other ccCoVs. For the DP0401 restricted ccCoV cell line generated with HKU-1 S_905-924_ peptide, the HKU-1 peptide elicited a stronger response compared to the corresponding SARS-CoV-2 peptide (S4C Fig). This result was confirmed by T cell proliferation assays in which HKU-1 peptide could elicit stronger responses at lower dosage compared to the SARS-CoV-2 peptides (S4D Fig). Thus SARS-CoV-2 T cells have weaker affinity for ccCoV epitopes and vice versa.

**Fig 5:**
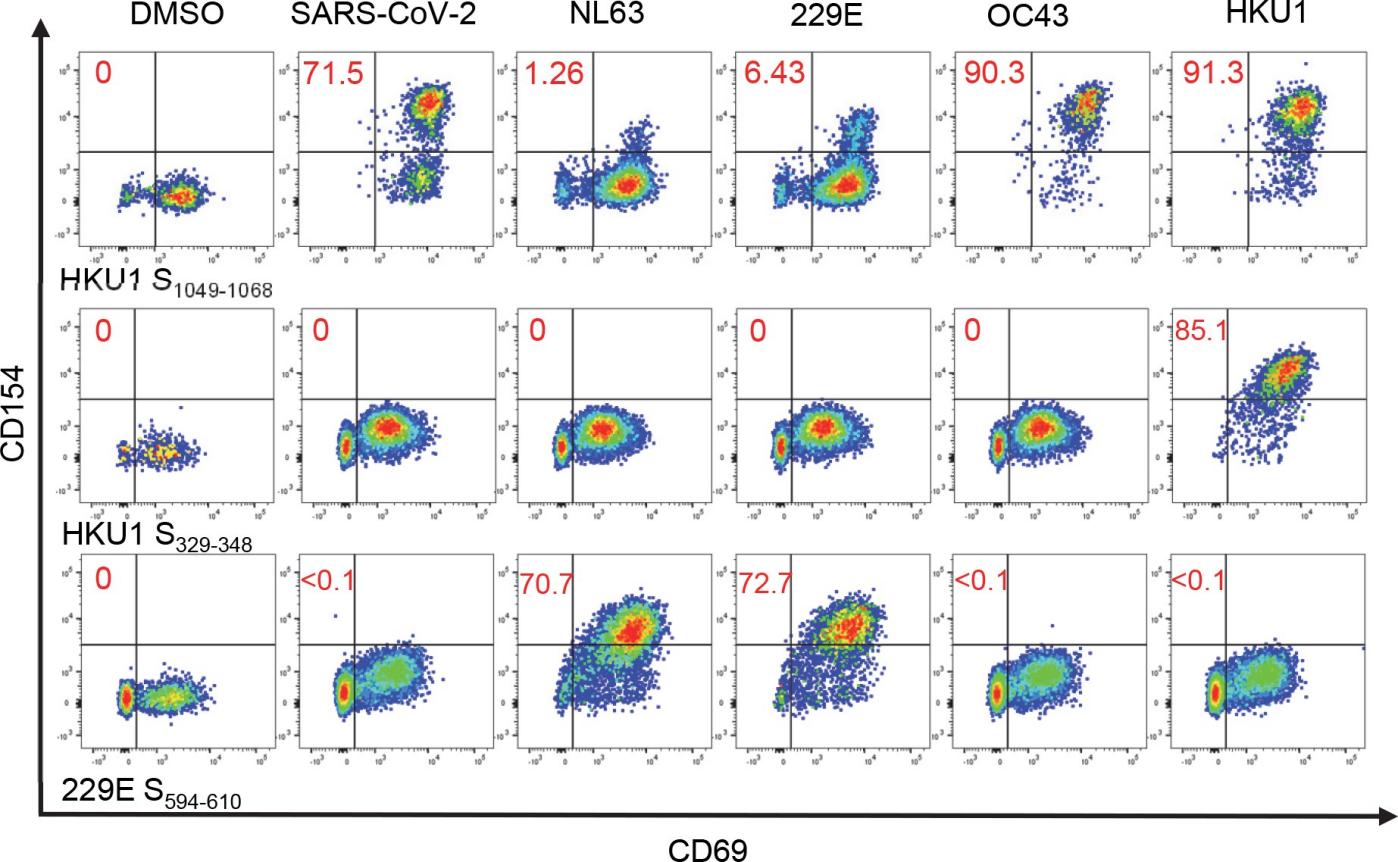
ccCoV Cross-reactive T Cells. CD154 upregulation assays of three potential cross-reactive ccCoV epitopes. HKU-1 S_1049-1068_ (top), HKU-1 S_329-348_ (middle), and 229E S_594-610_ (bottom). ccCoV-peptide-specific cell lines from nonexposed individuals were generated and activated with peptides for five hours (as listed in Table 3). Cells were stained with anti-CD154 and anti-CD69. Numbers in red indicate percentage of CD154+CD69+ T cells. DMSO was used as a negative control.

For evaluation of whether ccCoV T cells could cross-recognize SARS-CoV-2 with minimum AA sequence identity within the T cell epitope region, tetramer guided epitope mapping was used to identify DR0401 restricted S-specific epitopes of ccCoV. A total of 24 ccCoV DR0401 restricted Spike specific cell lines, including four NL63 lines, seven 229E lines, seven OC43 lines and six HKU-1 lines were generated by sorting of ccCoV tetramer positive T cells. The CD154 upregulation assay was used to evaluate the cross-reactivity of these lines amongst other ccCoV and SARS-CoV-2 epitopes. Though cross-reactivity between ccCoV was observed in 10 out of the 24 T cell lines tested, only the HKU S_1049-1068_ T cell line show cross-reactivity with SARS-CoV-2 S_967-983_ (Table 3 and Fig 5). Almost all of the cross-reactivity amongst the ccCoV were between the different alpha-ccCoV or between the different beta-ccCoV that have ≥ 67% AA identity within the predicted minimum T cell epitope region. The predicted minimum HKU-1 T cell epitope within S_1049-1068_ also has 67% AA identity with SARS-CoV-2 S_967-983_ T cell epitope. The OC43 T cell line S_1058-1074_ obtained from another individual had identical minimal core T cell epitope as HKU-1 S_1049-1068_ and did not cross-recognize the SARS-CoV-2 S_967-983._ This result together with the different degree of cross-reactivity as observed with the DP0401 SARS-CoV-2 S_809-828_ lines highlighted the fine specificity of TCR in dictating degree of cross-reactivity for epitopes with high AA sequence homology. Overall, our data suggested that a very limited number of SARS-CoV-2 T cells are capable of recognizing ccCoV and vice versa.

**Table 3:** Potential DR0401 restricted ccCoV cross-reactive epitopes identified in CD154 assay. Red indicates cross-reactive epitopes. Bold and italic red indicates the putative MHC-II binding motif as predicted by NetMHCII pan 4.0. Shaded grey column indicates the cell line generated from PBMCs stimulated with listed epitope and ccCoV. NT: not tested.

**NL63**	**229E**	**OC43**	**HKU-1**	**SARS-CoV-2**
293–309 VDVMRYNLNFSANSLDN	115–131 DVIRYNLNFEENLRRGT	264–280 NGFTLEYWVTPLTSRQY	249–268 TDNETLQYWVTPLSKRQYLL	260–276 AGAAAYYVGYLQPRTFL
908–924 AQYYNGIMVLPGVADAE	726–742 CAQYYNGIMVLPGVADA	947–963 CVQSYKGIKVLPPLLSE	937–956 DLLCVQSFNGIKVLPPILSE	848–864 DLICAQKFNGLTVLPPL
956–972 ARLNYVALQTDVLQENQ	773–789 IQARLNYVALQTDVLQE	994–1010 VQYRINGLGVTMDVLSQ	985–1004 LNVQYRINGLGVTMDVLNKN	904–920 YRFNGIGVTQNVLYENQ
1151–1167 GIYGYVLRQPNLVLYSD	973–989 GYVLRQPNLALYKEGNY	1177–1193 GDRGIAPKSGYFVNVNN	1169–1188 SGDVGIAPKQGYFIKHNDHW	1086–1102 KAHFPREGVFVSNGTHW
				
**229E**	**NL63**	**OC43**	**HKU-1**	**SARS-CoV-2**
55–71 NNWFLLTNTSSVVDGVV	229–245 GFPFNNWFLLTNGSTLV	NT	NT	197–213 IDGYFKIYSKHTPINLV
463–479 SNDTFLNGITYTSTSGN	645–661 NQSLAGGITYVSNSGNL	647–663 NATYYNSWQNLLYDSNG	641–660 VYYNSWQNLLYDSNGNIIGF	554–570 ESNKKFLPFQQFGRDIA
594–610 VEYLQITSTPIVVDCST	771–787 TSVQVEYLQITSTPIVV	809–825 MEEFIQTSSPKVTIDCA	801–820 VGQEEFIQTNSPKVTIDCSL	722–738 VTTEILPVSMTKTSVDC
726–742 CAQYYNGIMVLPGVADA	908–924 AQYYNGIMVLPGVADAE	947–963 CVQSYKGIKVLPPLLSE	937–956 DLLCVQSFNGIKVLPPILSE	848–864 DLICAQKFNGLTVLPPL
773–789 IQARLNYVALQTDVLQE	956–972 ARLNYVALQTDVLQENQ	994–1010 VQYRINGLGVTMDVLSQ	985–1004 LNVQYRINGLGVTMDVLNKN	904–920 YRFNGIGVTQNVLYENQ
973–989 GYVLRQPNLALYKEGNY	1151–1167 GIYGYVLRQPNLVLYSD	1177–1193 GDRGIAPKSGYFVNVNN	1169–1188 SGDVGIAPKQGYFIKHNDHW	1086–1102 KAHFPREGVFVSNGTHW
				
**OC43**	**HKU-1**	**NL63**	**229E**	**SARS-CoV-2**
49–65 LGTYYVLDRVYLNTTLF	41–60 DVSYGLGTYYILDRVYLNTT	43–59 LLPTHWFCANQSTSVYS	NT	29–45 TNSFTRGVYYPDKVFRS
91–107 LWFKPPFLSDFINGIFA	89–108 WYQKPFLSDFNNGIFSRVKN	NT	NT	78–94 RFDNPVLPFNDGVYFAS
222–238 GGTFYAYFTDTGVVTKF	201–220 HFYQERGTFYAYYADSGMPT	241–257 GSTLVDGVSRLYQPLRL	61–77 TNTSSVVDGVVRSFQPL	NT
276–292 TSRQYLLAFNQDGIIFN	257–276 WVTPLSKRQYLLKFDNRGVI	311–327 KSGVIVFKTLQYDVLFY	127–143 LRRGTILFKTSYGVVVF	239–255 QTLLALHRSYLTPGDSS
342–358 NIEAWLNDKSVPSPLNW	329–348 IDKWLNNFNVPSPLNWERKI	382–398 GQFYINGFKYFDLGFIE	199–215 GHFYINGYRYFTLGNVE	337–353 PFGEVFNATRFASVYAW
689–705 AAFHANSSEPALLFRNI	681–700 AAFHQNASSLALLYRNLKCS	681–697 PDQVAVYQQSIIGAMTA	499–515 PPDQLVVYQQAVVGAML	596–612 SVITPGTNTSNQVAVLY
1058–1074 LSNRFGAISASLQEILS	1049–1068 QQLFNKFGAISSSLQEILSR	1028–1044 LTSQLRHNFQAISNSIQ	849–865 SQLRQNFQAISSSIQAI	848–864 DLICAQKFNGLTVLPPL
				
**HKU-1**	**OC43**	**NL63**	**229E**	**SARS-CoV-2**
33–52 PRISEYVVDVSYGLGTYYIL	37–53 PISTDTVDVTNGLGTYY	31–47 GVPDNSSTIVTGLLPTH	NT	22–38 TQLPPAYTNSFTRGVYY
169–188 SRNESWHFDKSEPLCLFKKN	186–202 RKELWHLDTGVVSCLYK	217–233 IFSVQQDGRIPNGFPFN	37–53 ENVFAVESGGYIPSDFA	169–185 EYVSQPFLLMDLEGKQGN
329–348 IDKWLNNFNVPSPLNWERK	342–358 NIEAWLNDKSVPSPLNW	382–398 GQFYINGFKYFDLGFIE	199–215 GHFYINGYRYFTLGNVE	337–353 PFGEVFNATRFASVYAW
801–820 VGQEEFIQTNSPKVTQDCSL	809–825 MEEFIQTSSPKVTIDCA	771–787 TSVQVEYLQITSTPIVV	594–610 VEYLQITSTPIVVDCST	722–738 VTTEILPVSMTKTSVDC
1049–1068 QQLFNKFGAISSSLQEILSR	1058–1074 LSNRFGAISASLQEILS	1034–1050 HNFQAISNSIQAIYDRL	849–865 SQLRQNFQAISSSIQAI	967–983 SSNFGAISSVLNDILSR
1105–1124 FGAALAMEKVNECVKSQSPR	1111–1127 KFSAAQAMEKVNECVKS	1086–1102 GSRRLAQQKINECVKSQ	907–923 RQLAQQKVNECVKSQSK	1023–1039 NLAATKMSECVLGQSKR

**Fig 6:**
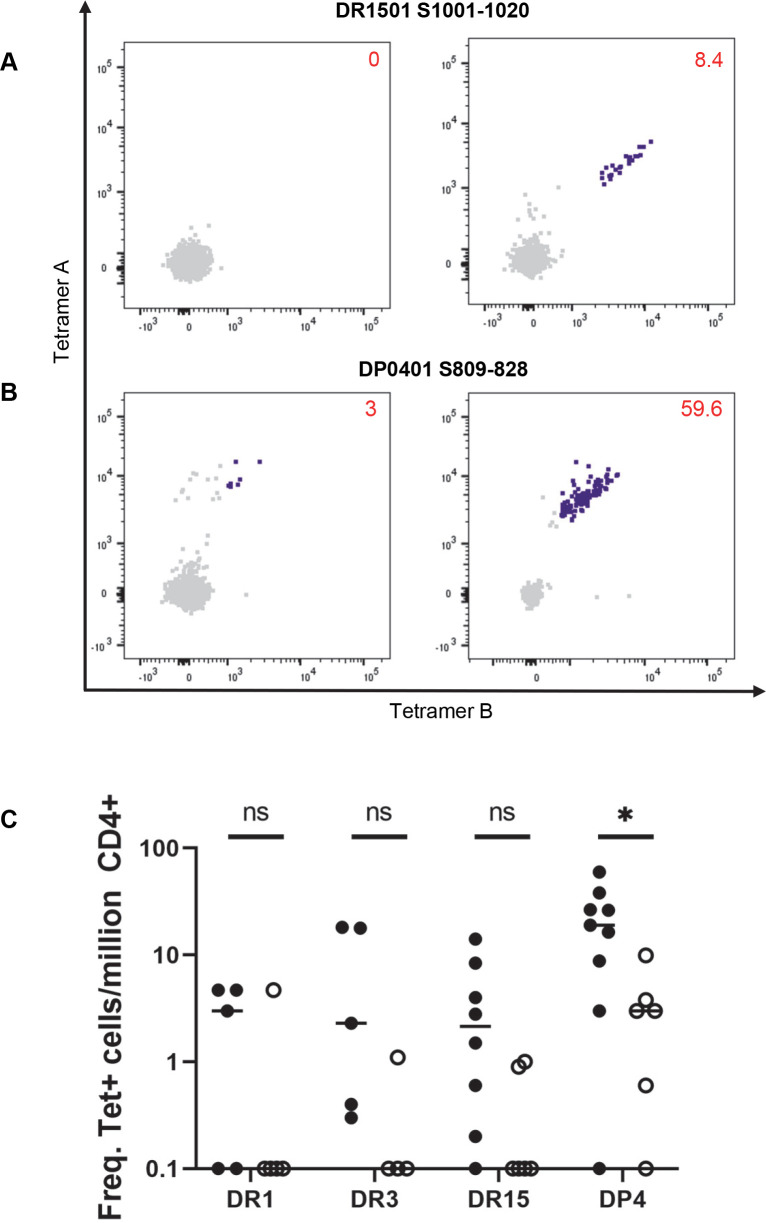
Frequencies of cross-reactive T cells. (A) *Ex vivo* staining of PBMC from DRB1501 individuals with tetramers. Representative FCS plots of PBMC from unexposed (left) and exposed (right) individuals stained with DR1501 SARS-CoV-2 S_1001-1020_-specific tetramers. Numbers indicate frequency of total tetramer positive cells per million CD4+ T cells. (B) *Ex vivo* staining of PBMC from DP0401 individuals with tetramers. Representative FCS plots of PBMC from unexposed (left) and exposed (right) individuals stained *ex vivo* with DP0401 SARS-CoV-2 S_809-828_-specific tetramers. Numbers indicate frequency of total tetramer positive cells per million CD4+ T cells. (C) Summary of total frequency of potential cross-reactive epitopes in *ex vivo* tetramer staining of PBMC in exposed (filled circle) and unexposed (open circle). The epitopes are: DR0101 SARS-CoV-2 S_961-980_, DR0301 SARS-CoV-2 S_985-964_, DR1501 SARS-CoV-2 S_1001-1020_, and DP0401 SARS-CoV-2 S_809-828_. Student’s unpaired t-test: *p≤0.05, ns = not significiant.

For examining the frequencies of T cells that recognized these S-specific cross-reactive epitopes, direct staining of PBMC from exposed persons and unexposed controls with tetramers was performed. Examples of direct staining and the summarized results are shown in Fig 6A–6C. These T cells were present at very low to undetectable frequencies in the unexposed group and their frequencies were elevated in exposed persons. With the exception of DP0401 S_809-828_, the average frequencies of these epitopes were less than 10 per million CD4+ T cells in exposed persons, indicating that most of these potential cross-reactive epitopes were incapable of eliciting a robust T cell immune response.

**Fig 7:**
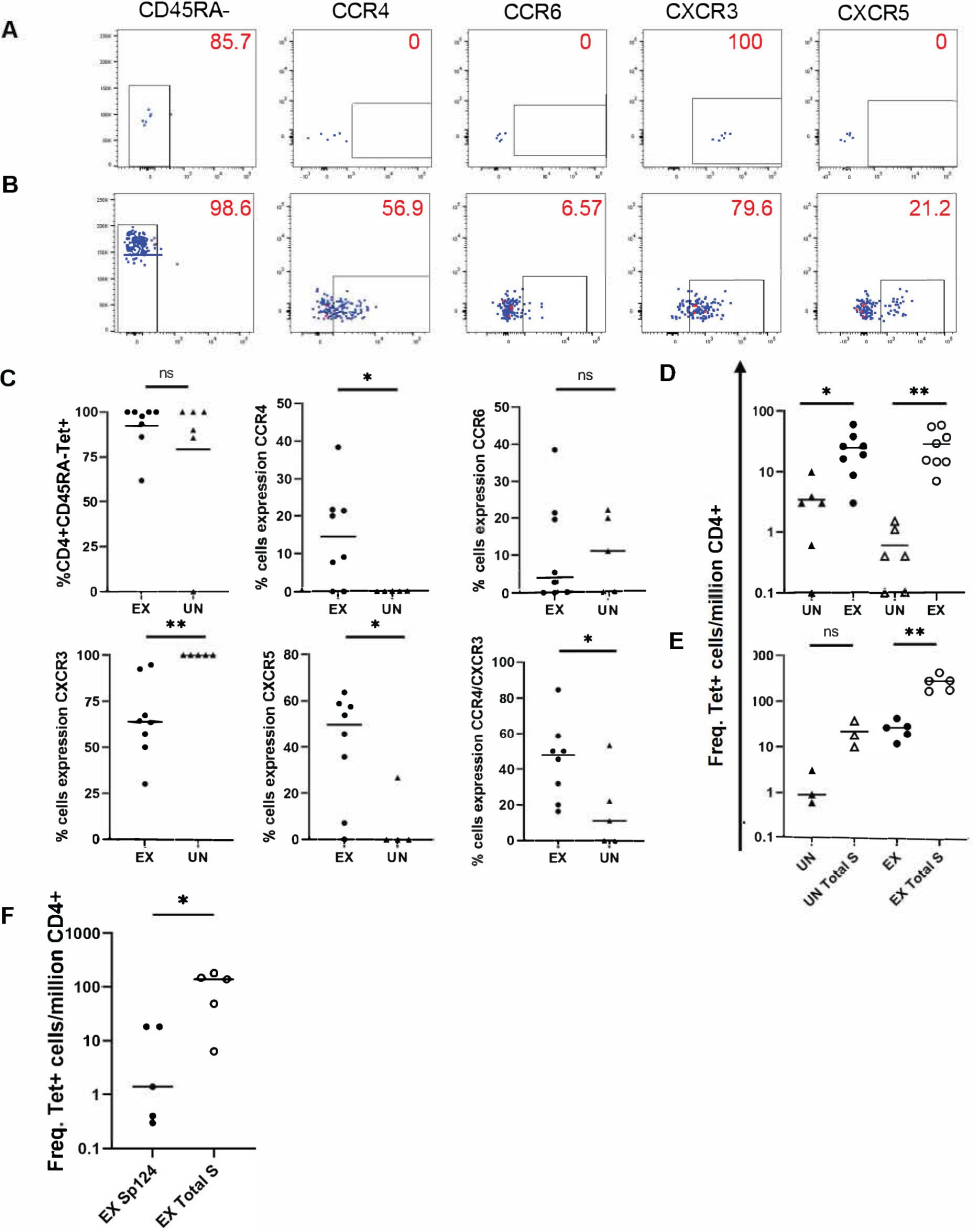
Comparison of DR1501- and DP0401-specific epitope in SARS-CoV-2 exposed and unexposed individuals. (A) Phenotype of DP0401 SARS-CoV-2 S_809-828_ cells stained *ex vivo* with DP0401 SARS-CoV-2 S_809-828_-specific tetramers in an unexposed individual. Numbers indicate percentage of cells with the corresponding surface marker. (B) Phenotype of DP0401 SARS-CoV-2 S_809-828_ cells stained *ex vivo* with DP0401 SARS-CoV-2 S_809-828_-specific tetramers in an exposed individual. Numbers indicate percentage of cells with the corresponding surface marker. (C) Summary of memory (top left) and phenotypes for all DP0401 SARS-CoV-2 S_809-828_ specific cells in exposed (ex) and unexposed (un) individuals. (D) Total frequency of DP0401 SARS CoV-2 S_809-828_ (closed circle and triangle) and DP0401 SARS-CoV-2 S_161-180_ (open circle and triangle) specific cells in exposed (ex) and unexposed (un) individuals. (E) Summary of combined DR1501 SARS-CoV-2 S_1001-1020_ and DP0401 SARS-CoV-2 S_809-828_ specific cells in individuals with DR1501/DRB5/DP0401 haplotype in *ex vivo* staining experiments compared to summed spike epitopes (total S) specific cells in exposed (ex) and unexposed (un) individuals. (F) Total frequency of DR0301 SARS-CoV-2 S_985-1004_ cells (closed circle) compared to the frequency of all summed spike epitopes (open circle) for DR0301 in exposed individuals. Student’s unpaired t-test for Figs 7A–7E and paired t-test of Fig 7E and 7F; * p≤0.05, ** p≤0.01, *** p≤0.001.

The phenotypes of the DP0401 T cells in unexposed and exposed persons were also examined. Examples of these staining and a summary of this data are shown in (Fig 7A, 7B, and 7C, respectively). The majority of the DP0401 SARS-CoV-2 S_809-828_ reactive T cells in the unexposed persons were memory T cells, implicating that these were T cells generated by previous ccCoV infections. In addition, a higher percentage of these cross-reactive cells in the COVID-19-convalescent group co-expressed CCR4 and CXCR3 compared to the SARS-CoV-2-unexposed group.

The extent of T cell expansion of the cross-reactive DP0401 SARS-CoV-2 S_809-828_ T cells was compared to the mono-reactive DP0401 SARS-CoV-2 S_161-180_ T cells. The frequency of the cross-reactive DP0401 SARS-CoV-2 S_809-828_ T cells was higher than the mono-reactive DP0401 SARS-CoV-2 S_161-180_ T cells in unexposed individuals (Fig 7D). However, the mean frequency of these cross-reactive and mono-reactive T cells was very similar in exposed subjects. On average, there was a 7-fold and 51-fold expansion of the cross-reactive DP0401 SARS-CoV-2 S_809-828_ and mono-reactive DP0401 SARS-CoV-2 S_161-180_ T cells, respectively. A similar comparison of the cross-reactive DR1501 SARS-CoV-2 S_1001-1020_ and the mono-reactive DR1501 SARS-CoV-2 S_689-708_/S_745-764_ T cells indicated a 9-fold and 53-fold expansion, respectively (Figs 6A and S5, respectively).

In order to evaluate the contribution of the cross-reactive S-specific CD4+ T cell responses to the overall S-specific CD4+ T cell responses in persons with DR1501-DRB5-DP401 haplotype, the frequencies of the sum of DP0401 SARS-CoV-2 S_809-828_- and DR1501 SARS-CoV-2 S_1001-1020_- cross-reactive T cells and the total S-specific T cell responses restricted by DR1501, DRB5, and DP0401 molecules in 5 exposed individuals with the DR1501-DRB5-DP0401 haplotype were examined. These data showed that the cross-reactive T cells contributed approximately 10% of the total S-specific T cell responses as presented by this haplotype (Fig 7E). Similar analysis shows that the DR0301 SARS-CoV-2 S_985-1004_ cross-reactive T cells contributed approximately 3% of the total S-specific T cell responses in subjects with the DR0301 allele (Fig 7F).

## Discussion

Though a large number of class II-restricted SARS-CoV-2 epitopes have been reported, a majority of these epitopes have not had the HLA restriction element defined. In this current report, class II tetramer reagents were used to identify CD4+ T cells epitopes. Both peptide epitopes and HLA restricting alleles were precisely determined using tetramer-guided epitope mapping. We also used tetramer reagents in *ex vivo* staining to show that SARS-CoV-2-unexposed persons harbor a minority of SARS-CoV-2-reactive CD4+ T cells suggesting that there is some cross-reaction between seasonal ccCoV and the pandemic virus. These findings have significance in understanding the spectrum of disease outcomes upon SARS-CoV-2 infection and in potentially stratifying immune responses to spike protein-based SARS-CoV-2 vaccines.

The epitope identification studies show that the structural proteins of SARS-CoV-2 are highly immunogenic in eliciting T cell responses. For the S protein, of the 158 peptides screened, 66 peptides were found to be immunogenic using a set of common class II alleles. Multiple epitopes can be identified for each HLA allele under study, with a mean frequency of 10 different S epitopes per allele. The T cell epitopes are almost evenly distributed along the S protein (S6 Fig). A person with two different DRB1 alleles and additional secondary DR, DQ and DP alleles should have more than 25 epitopes along the entire S protein and generate broad T cell repertories against the SARS-CoV-2 virus. We speculated that wide T cell repertories that are directed against the earlier SARS-CoV-2 strains, through either infection or vaccination, should be able to mount a significant T cell response against newly emergent SARS-CoV-2 variants. Indeed a recent report did show that COVID-19 convalescent individuals infected in the early phase of the pandemic mounted a robust CD4+ and CD8+ T cell responses against these new variants [42].

*Ex vivo* staining was performed to determine the frequencies of these epitope-specific T cells. For convalescent subjects, the mean frequencies of these cells were around 110, 54, and 63 per million CD4+ T cells for S, N, and M, respectively (Fig 3A). For unexposed subjects, the mean frequencies of S, N and M were 9, 1 and 8 per million CD4+ T cells respectively. The difference in frequencies represented a vast expansion of SARS-CoV-2 reactive T cells in infected people after exposure. T cell frequencies for a single epitope as high as between 100–200 per million CD4+ T cells were observed in some persons even months after infection. These experiments also show that all epitopes identified are not equal, as some T cell epitopes were more immunogenic compared to others (Figs 1 and S3). This should be taken into consideration in the evaluation of cross-reactive epitopes, as the presence of pre-existing T cells that recognize subdominant cross-reactive epitopes will have a limited effect on the overall T cell responses toward SARS-CoV-2. The frequencies of antigen-specific T cells as detected by tetramers in the current assay was much lower compared to those reported by the AIM assays but is in the range of those assayed by ELISPOT [8,10,13,14,16,17]. Notably, the tetramer assay focuses on a single HLA allele, while the AIM assay detects responses restricted by multiple HLA class II alleles within that person. Furthermore, the background signals of the AIM assays were as high as 0.016 to 0.042% of CD4+ T cell [8,10] compared to a staining background of less than 1 cell per million CD4+ T cells for each protein in HLA mismatch individual in the tetramer study.

Amongst the 66 S antigenic peptides identified in this study, 50 of the peptides identified have ≥67% AA sequence identity between SARS-CoV-2 and SARS-CoV-1 in the core epitope region, with 18 of these epitopes having 100% sequence identity (S4 Table). This comparison suggests that T cell cross-reactivity between SARS-CoV-2 and SARS-CoV-1 should be extensive. In contrast, only 4 of the SARS-CoV-2 S antigenic epitopes have more than 67% sequence identical with ccCoV in the core MHC binding region. All of these potential S reactive cross-reactive epitopes are located in the S2 region of the S protein.

We demonstrated that DR0301 SARS-CoV-2 S_985-1004_, DR1501 SARS-CoV-2 S_1001-1020_, and DP0401 SARS-CoV-2 S_809-828_ reactive T cells show functional cross-reactivity to ccCoV. Interestingly, of the three DP0401 individuals tested, DP0401 SARS-CoV-2 S_809-828_ cell lines from one individual showed cross-reactivity mainly to OC43 and HKU-1 (beta ccCoV) (Fig 4A), the second individual showed cross-reactivity mainly to NL63 and 229E (alpha ccCoV) (Fig 4C), while the third individual showed cross-reactivity to all four ccCoV (Fig 4B). These results show that the T cell repertoire of the individual or the individual’s previous exposure to ccCoV would dictate the nature of the cross-reactivity. The DR0101 SARS-CoV-2 S_961-980_ cell line generated in this study did not show cross-reactivity. We do expect SARS-CoV-2 S_961-980_ to be a cross-reactive epitope, as a DR0401 HKU-1 S_1049-1068_ cell line did cross recognize the SARS-CoV-2 S_967-983_ epitope. All this data illustrated that the fine specificity of the TCR would determine the nature of cross-reactivity for epitopes with high degree of AA identity, and it is likely that other DR0101 SARS-CoV-2 S_961-980_ should show cross-reactivity to HKU-1 S_1049-1068._

*Ex vivo* tetramer staining show DR0101 SARS-CoV-2 S_961-980,_ DR0301 SARS-CoV-2 S_985-1004_ and DR1501 SARS-CoV-2 S_1001-1020_ reactive T cells could not be consistently detected in unexposed individuals, suggesting that these are not immunodominant epitopes. It is unlikely the SARS-CoV-2 S_961-980_ is a dominant DR0401 restricted epitope, as T cells specific for this peptide cannot be detected in the TGEM studies in DR0401 COVID-19 subjects. In contrast, DP0401 SARS-CoV-2 S_809-828_ reactive T cells were detected in 5 out of 6 DP0401 unexposed individuals. Almost 100% of DP0401 SARS-CoV-2 S_809-828_ reactive T cells detected in unexposed individuals were CD45RA-CXCR3+, implicating that these are memory T cells from previous ccCoV infection. In addition to the increase in frequency of these cells post-infection, the phenotypes of these DP0401 cross-reactive T cells pre- and post-infection were also distinct. The cross-reactive T cells gained expression of CCR4 post SARS-CoV-2 infection, implying these cells are being activated for further expansion and differentiation during the disease process.

DP0401 is a prevalent allele and is estimated to be present in greater than 50% in both Europe and North America, and approximately in 30–40% of the world population, [37,43]. The DPB1*04:02 (DP0402) allele is also a prevalent allele, with phenotypic frequency similar to DP0401, has a similar peptide binding motif to DP0401 [43]. It was estimated that these two DP4 alleles together would cover 50–60% of the world population ([37,43]).Though not directly tested in the current work, it is likely that the DP0402 molecules can also present the SARS-CoV-2 S_809-828_ peptide. The prevalence of both DP0401 and DP0402 in the world population implies that a high percentage of the general population that had previous exposure to ccCoV should have these cross-reactive T cells. Interestingly, Low *et*. *al*., Dykema *et al*., and Woldemeskel *et* al [18,32,44] have also identified the presence of these DP4-restricted T cells by different approaches. Collectively, these data suggest a high prevalence of these DP4 cross-reactive T cells in the general population, and highlighted the potential roles of these cells in providing protection.

Despite these findings, frequencies of the DP0401 CoV-2 S_809-828_ reactive T cells as detected by tetramers in unexposed individuals in our cohort were still relatively low. The degree of expansion of the cross-reactive DP0401 SARS-CoV-2 S_809-828_ T cells after SARS-CoV-2 infection also appeared to be less vigorous compared to the mono-reactive DP0401 SARS-CoV-2 S_161-180_ epitopes. Less vigorous expansion of the cross-reactive DR1501 SARS-CoV-2 S_1001-1020_ T cells compared to another mono-reactive DR1501 SARS-CoV-2 S_689-708_ was also observed. It is unclear whether these cross-reactive TCRs have lower avidity to the MHC-II/SARS-CoV-2 peptide complexes compared to those of mono-reactive TCRs. We estimated that the cross-reactive DP0401 SARS-CoV-2 S_809-828_ and DR1501 SARS-CoV-2 S_1001-1020_ T cells contribute to less than 10% of the total Spike responses in DR1501-DP0401 individuals with no recent ccCoV infection. Similarly, the DR0301 SARS-CoV-2 S_985-1004_ contributed less than 3% of the overall S-restricted responses in subjects with the DR0301 haplotype.

The current observation of near absence or very low frequency of SARS-CoV-2 T cells in unexposed subjects as detected by tetramers was in contrast with most of the published data using the AIM assays in which cross-reactive T cells at relatively high frequencies were being detected in 20–60% of unexposed people. This difference in outcomes could be explained by the different assay being used.

A study showed SARS-CoV-2 T cells in unexposed subjects were 10–100 fold lower avidity compared to SARS-CoV-2 T cells in COVID-19 subjects [27]. The ability of the AIM assay to detect these low avidity T cells which escaped detection by tetramers probably account for the discrepancy in outcomes of these two different approaches. The question that remains to be resolved is whether both these low avidity and high avidity T cells can have a protective role in SARS-CoV-2 infection.

A limitation of this study is our focus on epitope specific T cells targeted toward the structural proteins of the virus. We do expect that the extent of cross-reactivity for T cells that targeted other regions of the virus should be very similar to the structural proteins as observed here, as degree of AA sequence homology between SARS-CoV-2 and ccCoV are fairly similar between the structural and non-structural proteins. Another limitation of our study is that tetramer reagents are incapable of detecting low avidity T cells. Though the use of CD154 upregulation assays with SARS-CoV-2 and ccCoV T cell in the current study also did not demonstrate a higher degree of cross-reactivity between SARS-CoV-2 and ccCoV compared to the tetramer approach. We acknowledge that ccCoV T cells specific for ccCoV epitope with AA sequence identity of <67% to SARS-CoV-2 could still potentially respond to SARS-CoV-2. Thus, sequence mismatches cannot completely rule out low avidity cross-reactivity. The current study did not investigate the prevalence of ccCoV T cells that had low avidity for SARS-CoV-2 in unexposed subjects. The relative absence of high avidity T cell cross-reactivity between ccCoV and SARS-CoV-2 as shown here also raise the possibility that the pre-existing SARS-CoV-2 T cells reported in other studies can be due to cross-reactivity between SARS-CoV-2 and other microbes. As it is known that T cell cross-reactivity can occur with minimum AA sequence homology [45–47].

In summary, we show that cross-reactive CD4+ T cells with high avidity for both SARS-CoV-2 and ccCoV as detected by tetramers are restricted to a very limited number of SARS-CoV-2 epitopes with AA sequence identity ≥67% between these viruses. Of the four Spike cross-reactive epitopes examined in more detail in the current study, only the dominant DP0401-restricted epitope was capable of eliciting a consistent T cell response in both unexposed and exposed persons. Though low avidity cross-reactive T cells may be prevalent, high avidity cross-reactivity that involves dominant SARS-CoV-2 epitopes is likely limited and will be restricted to persons with specific HLA alleles. As the structural proteins of SARS-CoV-2 are highly immunogenic and have multiple mono-reactive dominant epitopes, the contribution of high avidity cross-reactive CD4+ T cells to the overall SARS-CoV-2 specific T cell responses for individuals with no recent ccCoV infection may be minimal. The extent of protection that can be offered by these low and high avidity cross-reactive T cells warrants further studies.

## Materials and methods

### Ethics statement

The study was approved by Benaroya Research Institute and University of Washington Institutional Review Boards and all blood samples were obtained with written informed consent from the participants.

### Study cohort

A total of 34 COVID-19-convalsecent subjects were recruited between April 2020 and April 2021. Subjects were recruited through Virginia Mason Hospital and University of Washington. Attributes of these subjects are described in S1 Table. All COVID-19-convalescent subjects reported a positive PCR test for SARS-CoV-2 in the nasopharyngeal swab. In addition, 22 pre pandemic cryopreserved samples (samples collected before December 2019) were obtained from the Benaroya Research Institute Biorepository. The University of Washington cohort has been previously described [48,49]

HLA typing was performed with OLERUP SSP typing kit according to the manufacturer’s instruction or by sequencing at Scisco Genetics, Inc. (Seattle, WA).

### Tetramer reagents

The following class II monomers and multimers were produced for this study: DRA1/DRB1*01:01 (DR0101), DRA1/DRB1*03:01 (DR0301), DRA1/DRB1*04:01 (DR0401), DRA1/DRB1*04:04 (DR0404), DRA1/DRB1*07:01 (DR0701), DRA1/DRB1*11:01 (DR1101), DRA1/DRB1*11:04 (DR1104), DRA1/DRB1*15:01 (DR1501), DRA1/DRB3*01:01 (DRB3), DRA1/DRB4*01:01 (DRB4), DRA1/DRB5*01:01 (DRB5), and DPA1*0103/DPB1*04:01 (DP0401). Production of these molecules has been previously described [50,51]. Monomers were then cross-linked with label–streptavidin to form tetramer. With the exception of DP and DR1104 reagents, all other tetramer reagents have Myc-tag.

### Peptides

Peptide libraries for SARS-CoV-2 Spike (S, Accession: QIQ50192.1), Membrane (M, Accession: QIQ50195.1), and Nucleocapsid (N, Accession: QIQ50199.1) consisted of 20 amino acid (20-mers) long peptides with a twelve amino acid overlap. The S peptides consisted of 158 peptides. The M peptides consisted of 27 peptides. The N peptides consisted of 51 peptides. Peptide libraries for Spike of HKU-1 (Accession: YP_173238.1) were also 20-mers with a 12 amino acid overlap. Peptide libraries for Spike protein of NL63 (Accession: Q6Q1S2), 229E (Accession: NP_073551), and OC43 (Accession: NP_937950) cold viruses were 17-mers and were obtained from BEI Resources.

### Tetramer-guided epitope mapping

The tetramer-guided epitope mapping (TGEM) procedure was done as previously described [38,39]. Briefly, freshly isolated PBMCs from convalescent subjects were stimulated with S, M, and N peptide pools. In a 48-well plate, 4 million PBMC per well were stimulated with its corresponding peptide pool at 2μg/mL for each individual peptide (consisting of 10 peptides per pool) for 14 days with 10IU/mL of IL-2 added on day 6. After 14 days of stimulation, two aliquots of 100μl of resuspended cells were incubated with its corresponding pooled tetramer at 0.5mg/mL (consisting of 5 peptides per pool) for 45 minutes at 37°C. Cells were stained with CD3 FITC, CD4 BV421, and CD25 APC-Cy7 (S5 Table) and analyzed on a BD LSR II flow cytometer. Cells from pools that gave a positive signal were analyzed with tetramers containing the single peptides from that positive pool.

### Combinatorial *ex vivo* enrichment

The combinatorial *ex vivo* enrichment procedure was done as previously described [40]. Briefly, approximately 10–20 million cryopreserved PBMCs were thawed with benzonase nuclease added to thawing media (RPMI-40 media supplemented with 40% fetal bovine serum). PBMCs were resuspended in 200μl TCM and incubated with 50nM dasatinib for 10 minutes at 37°C. Cells were incubated with pooled tetramers for 100 minutes at room temperature. PBMCs were incubated with 20μl of anti-c-Myc or 40μl of anti-PE magnetic beads for 20 minutes at room temperature. A “pre-enriched” fraction was reserved for calculating the frequency and the remaining cells were enriched on a magnetic column following the manufacturer’s protocol. Flow through was retained for a second combinatorial tetramer panel staining following the steps above (S3 Table and S2A Fig) Pre-enriched and enriched cells were stained for 20 minutes at room temperature with CD14 FITC, CD19 FITC, CXCR5 BB700, CD4 V500, CCR4 BV605, CCR6 BV786, CXCR3 AF647, and CD45RA AF700 (S5 Table). Dead cells were detected by staining with 0.3μM Sytox Green.

Each sample was collected to completion on a BD LSR Fortessa flow cytometer. A schematic of the gating strategy is shown in S2B Fig. Data were analyzed with FloJo v.10.7.2 and GraphPad Prism 9. Frequencies of epitope-specific T cells per million CD4+ T cells were calculated using the following formula: F = (1,000,000 x tetramer-positive events from enriched tube)/(100 x live CD4+ T cell events in the pre-enriched tube).

### CD154 assay of spike cell lines

After TGEM, cells positive for a peptide that shared ≥67% sequence identity with ccCoV were harvested and stained with 0.5mg/mL of tetramer and incubated for 45 minutes at 37°C. Cells were stained with CD4 FITC and sorted at 20–40 cells per well in a 96-well round-bottom plate using a BD FACS Aria I. Cells were stimulated with 2μg/mL PHA and 10IU/mL IL-2 in the presence of irradiated feeder cells and expanded for 12–14 days in TCM. Cell lines were validated with corresponding tetramer and CD4 FITC. Cell lines were expanded until ≥700,000 cells were obtained with 10IU/mL IL-2 added every 2 days. Cells were rested for 5 days without the presence of IL-2 then resuspended and harvested. Cells were plated in a 96-well round-bottom plate at 1x10^5^ cells per well in TCM and 1 μg/mL anti-CD40 blocking antibody was added. Cells were incubated with 2μg/mL peptides or an equal volume of DMSO. After incubation, cells were resuspended, washed, and stained with CD3 FITC, CD4 PerCP-Cy5.5, CD69 BV650, and CD154 PE for 20 minutes at room temperature. Cell lines were analyzed on a BD LSR II flow cytometer with 50,000 events collected.

### Proliferation assay

T cells were plated at 5x10^4^ cells per well in a 96-well round-bottom plate, co-cultured with 1x10^5^ irradiated HLA-matched feeder cells and peptides from ccCoV and SARS-CoV-2 at concentrations of 2, 0.5 and 0.01μg/mL or DMSO (as negative control) for 72 hours. Cells were pulsed with 1μCi ^3^H-Thymidine for an additional 24 hours. Cells were harvested on Harvester 96 Mach II M. Uptake of ^3^H-Thymidine was measured on a Perkin Elmer MicroBeta2 scintillation counter to assess proliferation. Stimulation index (SI) was calculated by taking the average CCPM (corrected counts per minute) of peptide stimulation divided by the average CCPM of DMSO.

### Statistical analysis

GraphPad Prism 9 was used for data analysis.

## Supporting information

S1 Fig Tetramer-guided epitope mapping of DR0401 Spike pools. (A) Pool mapping of a representative DR0401 SARS-CoV-2-exposed individual with pools of overlapping peptides for the Spike protein. Bold FCS plots indicate positive pools. Numbers indicate percent of CD4+ Tetramer+ T cells. (B) Example of fine mapping of pools 1 and 24. Bolded FCS plots in **A**. Bold FCS plots indicate positive peptides. Positive peptides are S_25-44_, S_33-52_, S_929-948_, and S_937-956_.
(TIF)

S2 Fig Schematic of combinatorial *ex vivo* protocol and gating strategy. **(**A) 1. PBMCs were incubated with the first pool of tetramers conjugated to PE, PE-CF594, PE-Cy7, and BV421 for 100 minutes at room temperature following a 10 minute incubation with dasatinib at 37°C. 2. PBMCs were incubated with 40ul of anti-PE magnetic beads for 20 minutes at room temperature. 3. Tetramer-positive PBMCs were enriched on magnetic column. 4a. Tetramer-positive PBMCs were eluted from the column. 4b. Flow through of tetramer-negative PBMC was collected and incubated with second pool of tetramers and the process was repeated. 5. PBMCs were stained with antibody panel for 20 minutes at room temperature and then analyzed with flow cytometry. (B) Gating strategy to identify DRB1*04:01 HA306/HAp68 from an unexposed individual. Size gating was applied to select for singlet lymphocytes followed by a dump gate (CD14 FITC, CD19 FITC, and SYTOX Green) to exclude macrophages, B-cells, and dead cells. Live CD14-CD19- cells were gated for CD4+ cells. Live CD4+ cells were gated on the four tetramer fluorochromes (PE, PE-CF594, PE-Cy7, and BV421) and Boolean gating was applied to select for double-positive tetramer CD4+ cells. These double-positive cells were gated for memory cells on CD45RA-. Surface phenotypic markers were gated on CD45RA- memory cells.
(TIF)

S3 Fig Frequencies of T cells specific for dominant and subdominant Spike epitopes identified in *ex vivo* tetramer staining in three representative exposed individuals. (A) DR0301 dominant (D) and subdominant (S) epitopes. Open circle S_801-820_/S_857-876_; closed circle S_985-1004_/S_1073-1092_/S_1105-1124_; closed triangle S_345-364_/S_353-372_. (B) DR1501 dominant (D) and subdominant (S) epitopes. Open circle S_57-76_/S_97-116_; open triangle S_689-708_/S_745-764_; closed circle S_321-340_/S_425-444_. (C) DRB3 dominant (D) and subdominant (S) epitopes. Open circle S_25-44_/S_33-52_; open triangle S_209-228_/S_393-412_; closed triangle S_1249-1268_. (D) DP0401 dominant (D) and subdominant (S) epitopes. Open circle S_161-180_; open triangle S_809-828_; closed circle S_129-148_/S_337-356_.
(TIF)

S4 Fig CD154 upregulation assay of cell lines from unexposed individuals. PBMC of unexposed individuals were stimulated with ccCoV peptides and cultured for 14 days. Cold virus specific T cell lines from unexposed individuals were established by sorting of ccCoV tetramer positive T cells followed by expansion of sorted cells. CD154 upregulation assays were carried out, numbers indicate the percentage of CD154+CD69+ cells. (A) DR1501 OC43 S_1093-1109_ cell line. (B) DP0401 NL63 S_867-883_ cell line. (C) DP0401 HKU1 S_905-924_ cell line. (D) Proliferation assay on HKU1 S905-924 cell line generated from pre-pandemic DP0401 PBMC. SI: Stimulation index.
(TIF)

S5 Fig Dominant epitope DR1501 S_689-708_/S_745-764_ exposed and unexposed individuals. Representative FCS plot of ex vivo staining of T cells of dominant epitope S_689-708_/S_745-764_ in PBMC of unexposed (left) and exposed (middle) individuals. A summary of all individuals’ S_689-708_/S_745-764_ reactive T cell frequencies in DR1501 individuals is shown (left). Student’s unpaired t-test; ** p≤0.01.
(TIF)

S6 Fig Location of positive peptides identified in tetramer-guided epitope mapping (TGEM) and their position along the spike amino acid sequence. The above shows the Spike protein mutations in four prevalent SARS-CoV-2 strains, B.1.1.7, B.1.351, P.1, and B.1.617.2. Tan indicates the signal peptide, pink indicates S1, green RBD, and blue S2.
(TIF)

S1 Table Characteristic of the SARS-CoV-2 exposed and un-exposed individuals in this study. (DOCX)

S2 Table HLA-DR and DP allele frequency. (DOCX)

S3 Table Tetramer reagents used in combinatorial tetramer staining. (DOCX)

S4 Table SARS-CoV-2 Spike peptides identified in tetramer-guided epitope mapping (TGEM) with amino acid sequence identity to SARS-CoV-1. Red designated 100% AA identity to SARS-CoV-1. Green designated AA sequence with 1–3 AA mismatch to SARS CoV-1.
(DOCX)

S5 Table Antibody Reagents. (DOCX)

## References

[1] Zhu N, Zhang D, Wang W, Li X, Yang B, Song J, et al. A Novel Coronavirus from Patients with Pneumonia in China, 2019. N Engl J Med. 2020;382(8):727–33. doi: 10.1056/NEJMoa2001017 31978945 PMC7092803

[2] Salzberger B, Buder F, Lampl B, Ehrenstein B, Hitzenbichler F, Holzmann T, et al. Epidemiology of SARS-CoV-2. Infection. 2021;49(2):233–9. doi: 10.1007/s15010-020-01531-3 33034020 PMC7543961

[3] Williamson EJ, Walker AJ, Bhaskaran K, Bacon S, Bates C, Morton CE, et al. Factors associated with COVID-19-related death using OpenSAFELY. Nature. 2020;584(7821):430–6. doi: 10.1038/s41586-020-2521-4 32640463 PMC7611074

[4] Severe Covid GG, Ellinghaus D, Degenhardt F, Bujanda L, Buti M, Albillos A, et al. Genomewide Association Study of Severe Covid-19 with Respiratory Failure. N Engl J Med. 2020;383(16):1522–34. doi: 10.1056/NEJMoa2020283 32558485 PMC7315890

[5] Zhang Q, Bastard P, Liu Z, Le Pen J, Moncada-Velez M, Chen J, et al. Inborn errors of type I IFN immunity in patients with life-threatening COVID-19. Science. 2020;370(6515). doi: 10.1126/science.abd4570 32972995 PMC7857407

[6] Pairo-Castineira E, Clohisey S, Klaric L, Bretherick AD, Rawlik K, Pasko D, et al. Genetic mechanisms of critical illness in COVID-19. Nature. 2021;591(7848):92–8. doi: 10.1038/s41586-020-03065-y 33307546

[7] Zeberg H, Paabo S. The major genetic risk factor for severe COVID-19 is inherited from Neanderthals. Nature. 2020;587(7835):610–2. doi: 10.1038/s41586-020-2818-3 32998156

[8] Grifoni A, Weiskopf D, Ramirez SI, Mateus J, Dan JM, Moderbacher CR, et al. Targets of T Cell Responses to SARS-CoV-2 Coronavirus in Humans with COVID-19 Disease and Unexposed Individuals. Cell. 2020;181(7):1489–501 e15. doi: 10.1016/j.cell.2020.05.015 32473127 PMC7237901

[9] Weiskopf D, Schmitz KS, Raadsen MP, Grifoni A, Okba NMA, Endeman H, et al. Phenotype and kinetics of SARS-CoV-2-specific T cells in COVID-19 patients with acute respiratory distress syndrome. Sci Immunol. 2020;5(48). doi: 10.1126/sciimmunol.abd2071 32591408 PMC7319493

[10] Rydyznski Moderbacher C, Ramirez SI, Dan JM, Grifoni A, Hastie KM, Weiskopf D, et al. Antigen-Specific Adaptive Immunity to SARS-CoV-2 in Acute COVID-19 and Associations with Age and Disease Severity. Cell. 2020;183(4):996–1012 e19. doi: 10.1016/j.cell.2020.09.038 33010815 PMC7494270

[11] Dan JM, Mateus J, Kato Y, Hastie KM, Yu ED, Faliti CE, et al. Immunological memory to SARS-CoV-2 assessed for up to 8 months after infection. Science. 2021;371(6529). doi: 10.1126/science.abf4063 33408181 PMC7919858

[12] Sekine T, Perez-Potti A, Rivera-Ballesteros O, Stralin K, Gorin JB, Olsson A, et al. Robust T Cell Immunity in Convalescent Individuals with Asymptomatic or Mild COVID-19. Cell. 2020;183(1):158–68 e14. doi: 10.1016/j.cell.2020.08.017 32979941 PMC7427556

[13] Peng Y, Mentzer AJ, Liu G, Yao X, Yin Z, Dong D, et al. Broad and strong memory CD4(+) and CD8(+) T cells induced by SARS-CoV-2 in UK convalescent individuals following COVID-19. Nat Immunol. 2020;21(11):1336–45. doi: 10.1038/s41590-020-0782-6 32887977 PMC7611020

[14] Ogbe A, Kronsteiner B, Skelly DT, Pace M, Brown A, Adland E, et al. T cell assays differentiate clinical and subclinical SARS-CoV-2 infections from cross-reactive antiviral responses. Nat Commun. 2021;12(1):2055. doi: 10.1038/s41467-021-21856-3 33824342 PMC8024333

[15] Braun J, Loyal L, Frentsch M, Wendisch D, Georg P, Kurth F, et al. SARS-CoV-2-reactive T cells in healthy donors and patients with COVID-19. Nature. 2020;587(7833):270–4. doi: 10.1038/s41586-020-2598-9 32726801

[16] Tan AT, Linster M, Tan CW, Le Bert N, Chia WN, Kunasegaran K, et al. Early induction of functional SARS-CoV-2-specific T cells associates with rapid viral clearance and mild disease in COVID-19 patients. Cell Rep. 2021;34(6):108728. doi: 10.1016/j.celrep.2021.108728 33516277 PMC7826084

[17] Zuo J, Dowell AC, Pearce H, Verma K, Long HM, Begum J, et al. Robust SARS-CoV-2-specific T cell immunity is maintained at 6 months following primary infection. Nat Immunol. 2021;22(5):620–6. doi: 10.1038/s41590-021-00902-8 33674800 PMC7610739

[18] Low JS, Vaqueirinho D, Mele F, Foglierini M, Jerak J, Perotti M, et al. Clonal analysis of immunodominance and cross-reactivity of the CD4 T cell response to SARS-CoV-2. Science. 2021. doi: 10.1126/science.abg8985 34006597 PMC8168615

[19] Tarke A, Sidney J, Kidd CK, Dan JM, Ramirez SI, Yu ED, et al. Comprehensive analysis of T cell immunodominance and immunoprevalence of SARS-CoV-2 epitopes in COVID-19 cases. bioRxiv. 2020. doi: 10.1101/2020.12.08.416750 33521695 PMC7837622

[20] Nelde A, Bilich T, Heitmann JS, Maringer Y, Salih HR, Roerden M, et al. SARS-CoV-2-derived peptides define heterologous and COVID-19-induced T cell recognition. Nat Immunol. 2021;22(1):74–85. doi: 10.1038/s41590-020-00808-x 32999467

[21] Saini SK, Hersby DS, Tamhane T, Povlsen HR, Amaya Hernandez SP, Nielsen M, et al. SARS-CoV-2 genome-wide T cell epitope mapping reveals immunodominance and substantial CD8(+) T cell activation in COVID-19 patients. Sci Immunol. 2021;6(58). doi: 10.1126/sciimmunol.abf7550 33853928 PMC8139428

[22] Schulien I, Kemming J, Oberhardt V, Wild K, Seidel LM, Killmer S, et al. Characterization of pre-existing and induced SARS-CoV-2-specific CD8(+) T cells. Nat Med. 2021;27(1):78–85. doi: 10.1038/s41591-020-01143-2 33184509

[23] Nguyen THO, Rowntree LC, Petersen J, Chua BY, Hensen L, Kedzierski L, et al. CD8(+) T cells specific for an immunodominant SARS-CoV-2 nucleocapsid epitope display high naive precursor frequency and TCR promiscuity. Immunity. 2021;54(5):1066–82 e5. doi: 10.1016/j.immuni.2021.04.009 33951417 PMC8049468

[24] Kared H, Redd AD, Bloch EM, Bonny TS, Sumatoh H, Kairi F, et al. SARS-CoV-2-specific CD8+ T cell responses in convalescent COVID-19 individuals. J Clin Invest. 2021;131(5). doi: 10.1172/JCI145476 33427749 PMC7919723

[25] Mateus J, Grifoni A, Tarke A, Sidney J, Ramirez SI, Dan JM, et al. Selective and cross-reactive SARS-CoV-2 T cell epitopes in unexposed humans. Science. 2020;370(6512):89–94. doi: 10.1126/science.abd3871 32753554 PMC7574914

[26] Le Bert N, Tan AT, Kunasegaran K, Tham CYL, Hafezi M, Chia A, et al. SARS-CoV-2-specific T cell immunity in cases of COVID-19 and SARS, and uninfected controls. Nature. 2020;584(7821):457–62. doi: 10.1038/s41586-020-2550-z 32668444

[27] Bacher P, Rosati E, Esser D, Martini GR, Saggau C, Schiminsky E, et al. Low-Avidity CD4(+) T Cell Responses to SARS-CoV-2 in Unexposed Individuals and Humans with Severe COVID-19. Immunity. 2020;53(6):1258–71 e5. doi: 10.1016/j.immuni.2020.11.016 33296686 PMC7689350

[28] Coronaviridae Study Group of the International Committee on Taxonomy of V. The species Severe acute respiratory syndrome-related coronavirus: classifying 2019-nCoV and naming it SARS-CoV-2. Nat Microbiol. 2020;5(4):536–44. doi: 10.1038/s41564-020-0695-z 32123347 PMC7095448

[29] Chen Y, Liu Q, Guo D. Emerging coronaviruses: Genome structure, replication, and pathogenesis. J Med Virol. 2020;92(4):418–23. doi: 10.1002/jmv.25681 31967327 PMC7167049

[30] Lineburg KE, Grant EJ, Swaminathan S, Chatzileontiadou DSM, Szeto C, Sloane H, et al. CD8(+) T cells specific for an immunodominant SARS-CoV-2 nucleocapsid epitope cross-react with selective seasonal coronaviruses. Immunity. 2021;54(5):1055–65 e5. doi: 10.1016/j.immuni.2021.04.006 33945786 PMC8043652

[31] Woldemeskel BA, Kwaa AK, Garliss CC, Laeyendecker O, Ray SC, Blankson JN. Healthy donor T cell responses to common cold coronaviruses and SARS-CoV-2. J Clin Invest. 2020;130(12):6631–8. doi: 10.1172/JCI143120 32966269 PMC7685719

[32] Dykema AG, Zhang B, Woldemeskel BA, Garliss CC, Cheung LS, Choudhury D, et al. Functional characterization of CD4+ T-cell receptors cross-reactive for SARS-CoV-2 and endemic coronaviruses. J Clin Invest. 2021. doi: 10.1172/JCI146922 33830946 PMC8121515

[33] Sette A, Crotty S. Pre-existing immunity to SARS-CoV-2: the knowns and unknowns. Nat Rev Immunol. 2020;20(8):457–8. doi: 10.1038/s41577-020-0389-z 32636479 PMC7339790

[34] Lipsitch M, Grad YH, Sette A, Crotty S. Cross-reactive memory T cells and herd immunity to SARS-CoV-2. Nat Rev Immunol. 2020;20(11):709–13. doi: 10.1038/s41577-020-00460-4 33024281 PMC7537578

[35] Sagar M, Reifler K, Rossi M, Miller NS, Sinha P, White LF, et al. Recent endemic coronavirus infection is associated with less-severe COVID-19. J Clin Invest. 2021;131(1). doi: 10.1172/JCI143380 32997649 PMC7773342

[36] Meyer B, Drosten C, Muller MA. Serological assays for emerging coronaviruses: challenges and pitfalls. Virus Res. 2014;194:175–83. doi: 10.1016/j.virusres.2014.03.018 24670324 PMC7114385

[37] Hurley CK, Kempenich J, Wadsworth K, Sauter J, Hofmann JA, Schefzyk D, et al. Common, intermediate and well-documented HLA alleles in world populations: CIWD version 3.0.0. HLA. 2020;95(6):516–31. doi: 10.1111/tan.13811 31970929 PMC7317522

[38] Yang J, James EA, Huston L, Danke NA, Liu AW, Kwok WW. Multiplex mapping of CD4 T cell epitopes using class II tetramers. Clin Immunol. 2006;120(1):21–32. doi: 10.1016/j.clim.2006.03.008 16677863

[39] James EA, Bui J, Berger D, Huston L, Roti M, Kwok WW. Tetramer-guided epitope mapping reveals broad, individualized repertoires of tetanus toxin-specific CD4+ T cells and suggests HLA-based differences in epitope recognition. Int Immunol. 2007;19(11):1291–301. doi: 10.1093/intimm/dxm099 17906339

[40] Uchtenhagen H, Rims C, Blahnik G, Chow IT, Kwok WW, Buckner JH, et al. Efficient ex vivo analysis of CD4+ T-cell responses using combinatorial HLA class II tetramer staining. Nat Commun. 2016;7:12614. doi: 10.1038/ncomms12614 27571776 PMC5013714

[41] Reynisson B, Alvarez B, Paul S, Peters B, Nielsen M. NetMHCpan-4.1 and NetMHCIIpan-4.0: improved predictions of MHC antigen presentation by concurrent motif deconvolution and integration of MS MHC eluted ligand data. Nucleic Acids Res. 2020;48(W1):W449–W54. doi: 10.1093/nar/gkaa379 32406916 PMC7319546

[42] Tarke A, Sidney J, Methot N, Zhang Y, Dan JM, Goodwin B, et al. Negligible impact of SARS-CoV-2 variants on CD4 (+) and CD8 (+) T cell reactivity in COVID-19 exposed donors and vaccinees. bioRxiv. 2021. doi: 10.1101/2021.02.27.433180 34230917 PMC8249675

[43] Sidney J, Steen A, Moore C, Ngo S, Chung J, Peters B, et al. Five HLA-DP molecules frequently expressed in the worldwide human population share a common HLA supertypic binding specificity. J Immunol. 2010;184(5):2492–503. doi: 10.4049/jimmunol.0903655 20139279 PMC2935290

[44] Woldemeskel BA, Garliss CC, Blankson JN. SARS-CoV-2 mRNA vaccines induce broad CD4+ T cell responses that recognize SARS-CoV-2 variants and HCoV-NL63. J Clin Invest. 2021;131(10). doi: 10.1172/JCI149335 33822770 PMC8121504

[45] Quaratino S, Thorpe CJ, Travers PJ, Londei M. Similar antigenic surfaces, rather than sequence homology, dictate T-cell epitope molecular mimicry. Proc Natl Acad Sci U S A. 1995;92(22):10398–402. doi: 10.1073/pnas.92.22.10398 7479792 PMC40804

[46] Riley TP, Hellman LM, Gee MH, Mendoza JL, Alonso JA, Foley KC, et al. T cell receptor cross-reactivity expanded by dramatic peptide-MHC adaptability. Nat Chem Biol. 2018;14(10):934–42. doi: 10.1038/s41589-018-0130-4 30224695 PMC6371774

[47] Lee CH, Salio M, Napolitani G, Ogg G, Simmons A, Koohy H. Predicting Cross-Reactivity and Antigen Specificity of T Cell Receptors. Front Immunol. 2020;11:565096. doi: 10.3389/fimmu.2020.565096 33193332 PMC7642207

[48] Boonyaratanakornkit J, Morishima C, Selke S, Zamora D, McGuffin S, Shapiro AE, et al. Clinical, laboratory, and temporal predictors of neutralizing antibodies against SARS-CoV-2 among COVID-19 convalescent plasma donor candidates. J Clin Invest. 2021;131(3). doi: 10.1172/JCI144930 33320842 PMC7843229

[49] Phan IQ, Subramanian S, Kim D, Murphy M, Pettie D, Carter L, et al. In silico detection of SARS-CoV-2 specific B-cell epitopes and validation in ELISA for serological diagnosis of COVID-19. Sci Rep. 2021;11(1):4290. doi: 10.1038/s41598-021-83730-y 33619344 PMC7900118

[50] Novak EJ, Liu AW, Nepom GT, Kwok WW. MHC class II tetramers identify peptide-specific human CD4(+) T cells proliferating in response to influenza A antigen. J Clin Invest. 1999;104(12):R63–7. doi: 10.1172/JCI8476 10606632 PMC480919

[51] Yang J, Huston L, Berger D, Danke NA, Liu AW, Disis ML, et al. Expression of HLA-DP0401 molecules for identification of DP0401 restricted antigen specific T cells. JClinImmunol. 2005;25(5):428–36. doi: 10.1007/s10875-005-6095-6 16160911

