# Supplementary figures and images for: Cross-reactive and mono-reactive SARS-CoV-2 CD4+ T cells in prepandemic and COVID-19 convalescent individuals
Source: PLoS Pathog. 2021 Dec 29;17(12):e1010203. doi: 10.1371/journal.ppat.1010203 (PMC8769337; doi:10.1371/journal.ppat.1010203)

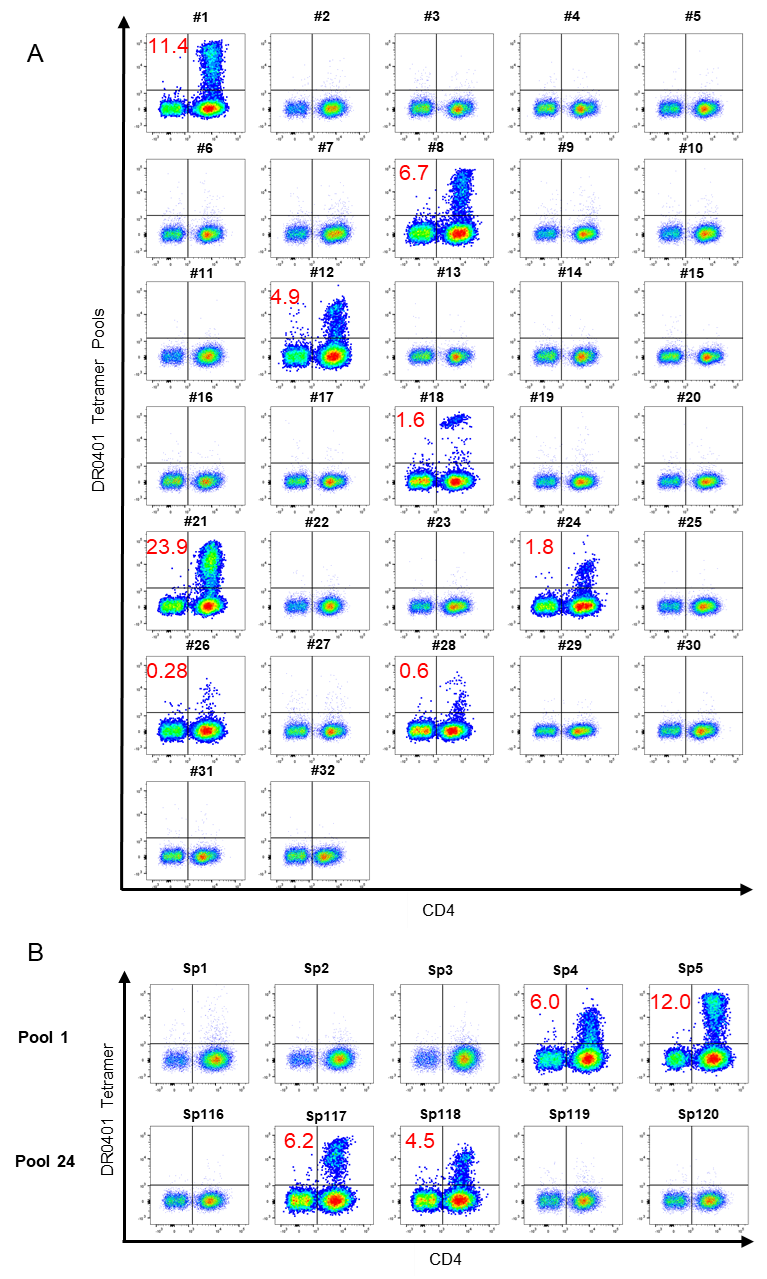

Supplement: S1 Fig — (A) Pool mapping of a representative DR0401 SARS-CoV-2-exposed individual with pools of overlapping peptides for the Spike protein. Bold FCS plots indicate positive pools. Numbers indicate percent of CD4+ Tetramer+ T cells. (B) Example of fine mapping of pools 1 and 24. Bolded FCS plots in A. Bold FCS plots indicate positive peptides. Positive peptides are S25-44, S33-52, S929-948, and S937-956. (TIF) [file ppat.1010203.s001.tif]

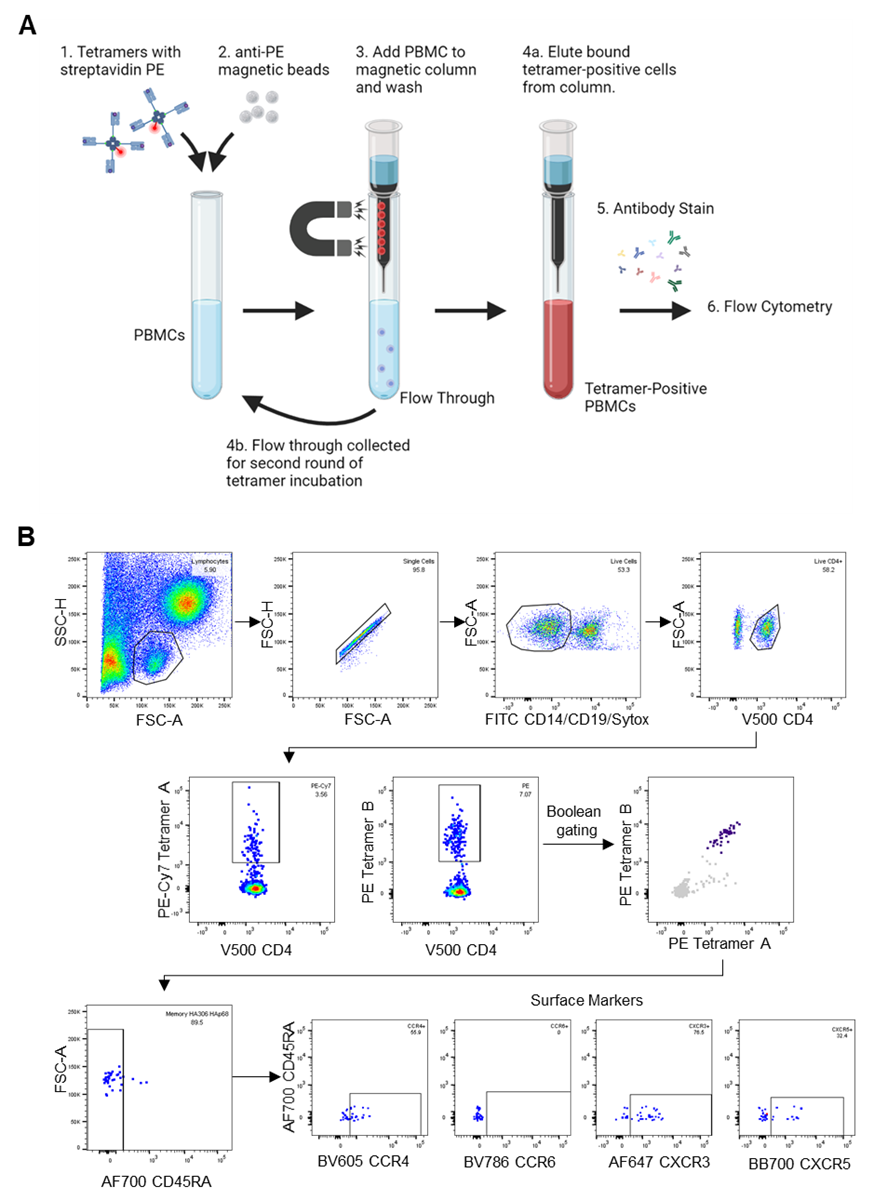

Supplement: S2 Fig — (A) 1. PBMCs were incubated with the first pool of tetramers conjugated to PE, PE-CF594, PE-Cy7, and BV421 for 100 minutes at room temperature following a 10 minute incubation with dasatinib at 37°C. 2. PBMCs were incubated with 40ul of anti-PE magnetic beads for 20 minutes at room temperature. 3. Tetramer-positive PBMCs were enriched on magnetic column. 4a. Tetramer-positive PBMCs were eluted from the column. 4b. Flow through of tetramer-negative PBMC was collected and incubated with second pool of tetramers and the process was repeated. 5. PBMCs were stained with antibody panel for 20 minutes at room temperature and then analyzed with flow cytometry. (B) Gating strategy to identify DRB1*04:01 HA306/HAp68 from an unexposed individual. Size gating was applied to select for singlet lymphocytes followed by a dump gate (CD14 FITC, CD19 FITC, and SYTOX Green) to exclude macrophages, B-cells, and dead cells. Live CD14-CD19- cells were gated for CD4+ cells. Live CD4+ cells were gated on the four tetramer fluorochromes (PE, PE-CF594, PE-Cy7, and BV421) and Boolean gating was applied to select for double-positive tetramer CD4+ cells. These double-positive cells were gated for memory cells on CD45RA-. Surface phenotypic markers were gated on CD45RA- memory cells. (TIF) [file ppat.1010203.s002.tif]

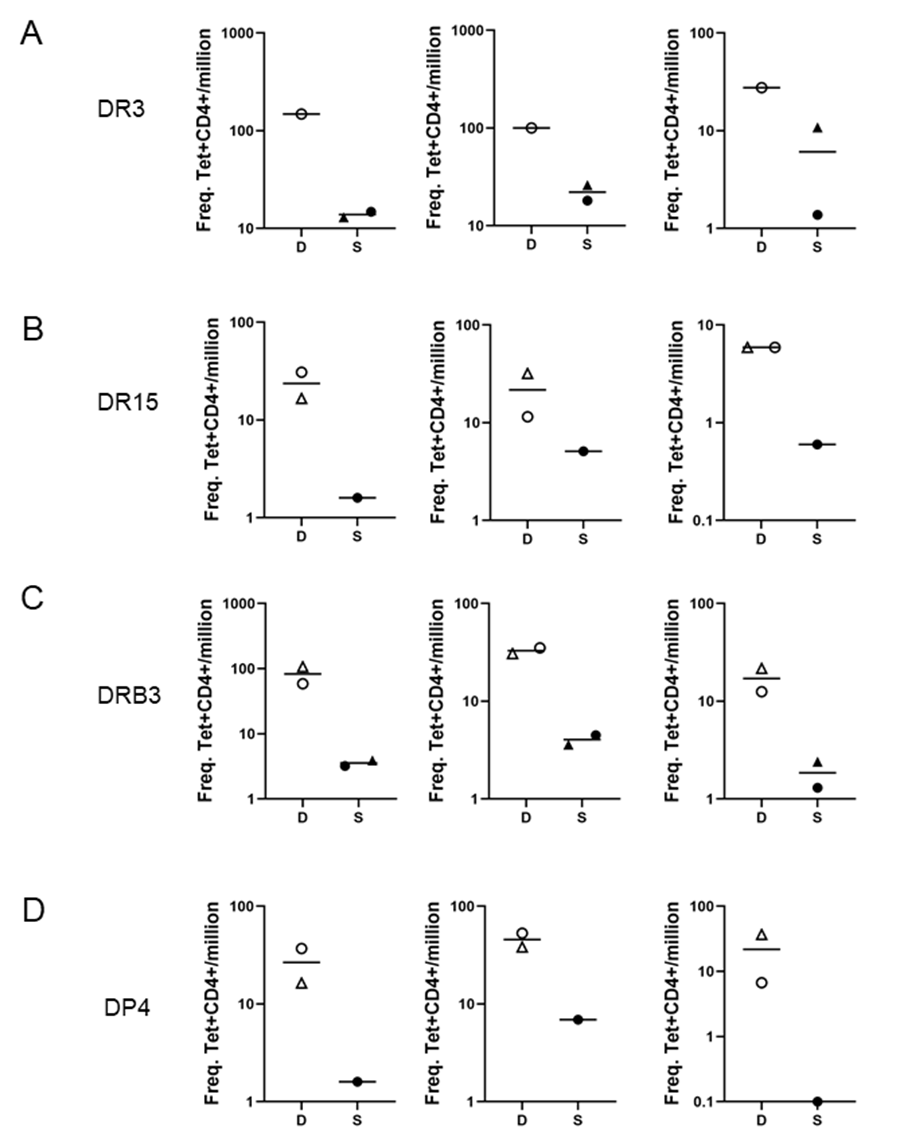

Supplement: S3 Fig — (A) DR0301 dominant (D) and subdominant (S) epitopes. Open circle S801-820/S857-876; closed circle S985-1004/S1073-1092/S1105-1124; closed triangle S345-364/S353-372. (B) DR1501 dominant (D) and subdominant (S) epitopes. Open circle S57-76/S97-116; open triangle S689-708/S745-764; closed circle S321-340/S425-444. (C) DRB3 dominant (D) and subdominant (S) epitopes. Open circle S25-44/S33-52; open triangle S209-228/S393-412; closed triangle S1249-1268. (D) DP0401 dominant (D) and subdominant (S) epitopes. Open circle S161-180; open triangle S809-828; closed circle S129-148/S337-356. (TIF) [file ppat.1010203.s003.tif]

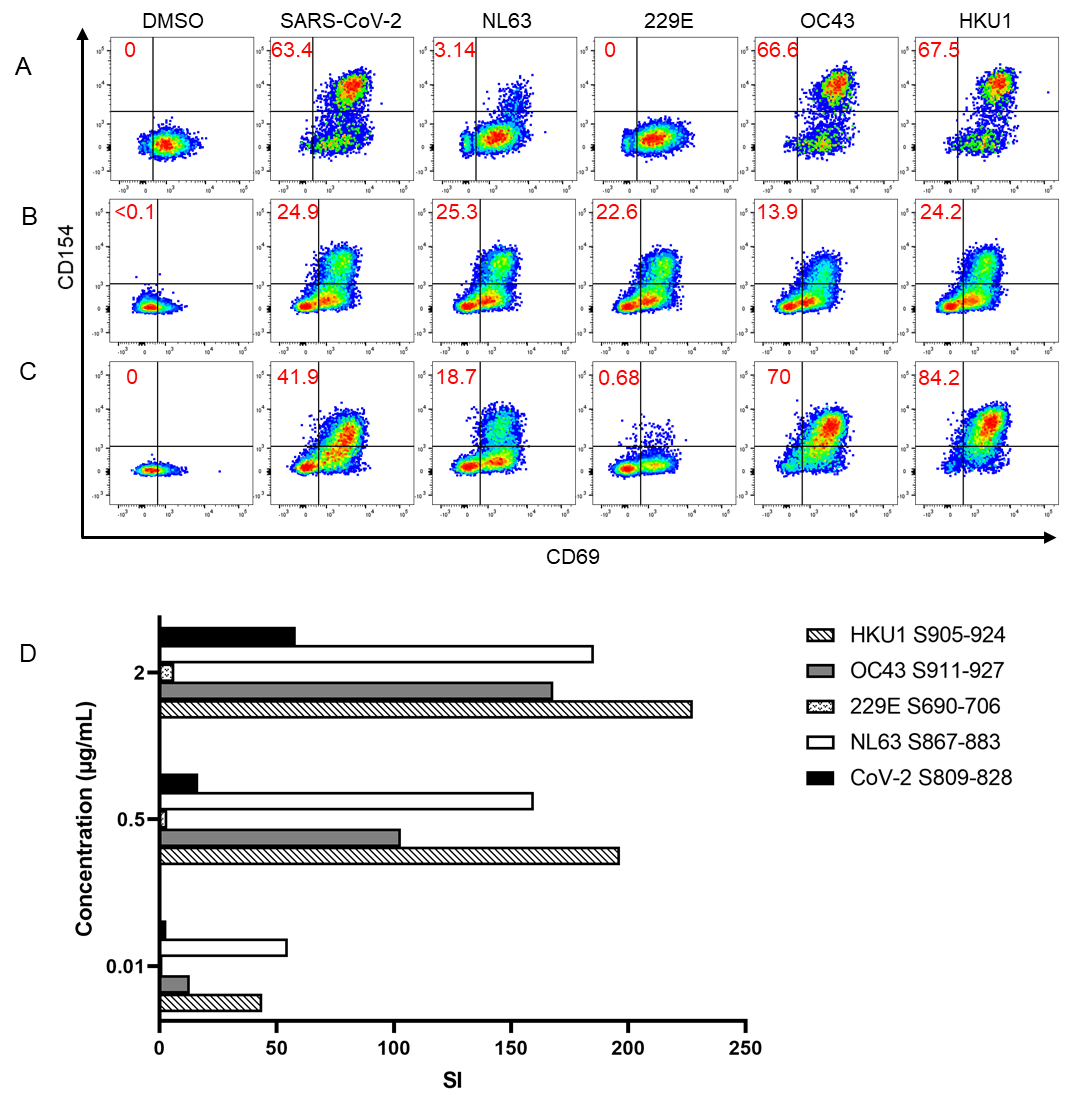

Supplement: S4 Fig — PBMC of unexposed individuals were stimulated with ccCoV peptides and cultured for 14 days. Cold virus specific T cell lines from unexposed individuals were established by sorting of ccCoV tetramer positive T cells followed by expansion of sorted cells. CD154 upregulation assays were carried out, numbers indicate the percentage of CD154+CD69+ cells. (A) DR1501 OC43 S1093-1109 cell line. (B) DP0401 NL63 S867-883 cell line. (C) DP0401 HKU1 S905-924 cell line. (D) Proliferation assay on HKU1 S905-924 cell line generated from pre-pandemic DP0401 PBMC. SI: Stimulation index. (TIF) [file ppat.1010203.s004.tif]

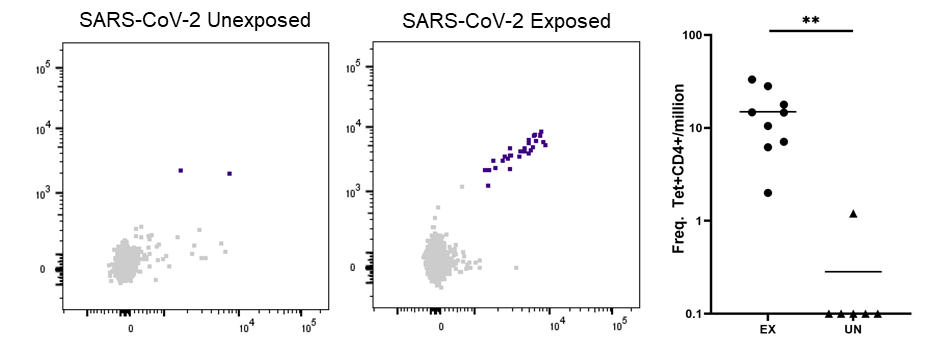

Supplement: S5 Fig — Representative FCS plot of ex vivo staining of T cells of dominant epitope S689-708/S745-764 in PBMC of unexposed (left) and exposed (middle) individuals. A summary of all individuals’ S689-708/S745-764 reactive T cell frequencies in DR1501 individuals is shown (left). Student’s unpaired t-test; ** p≤0.01. (TIF) [file ppat.1010203.s005.tif]

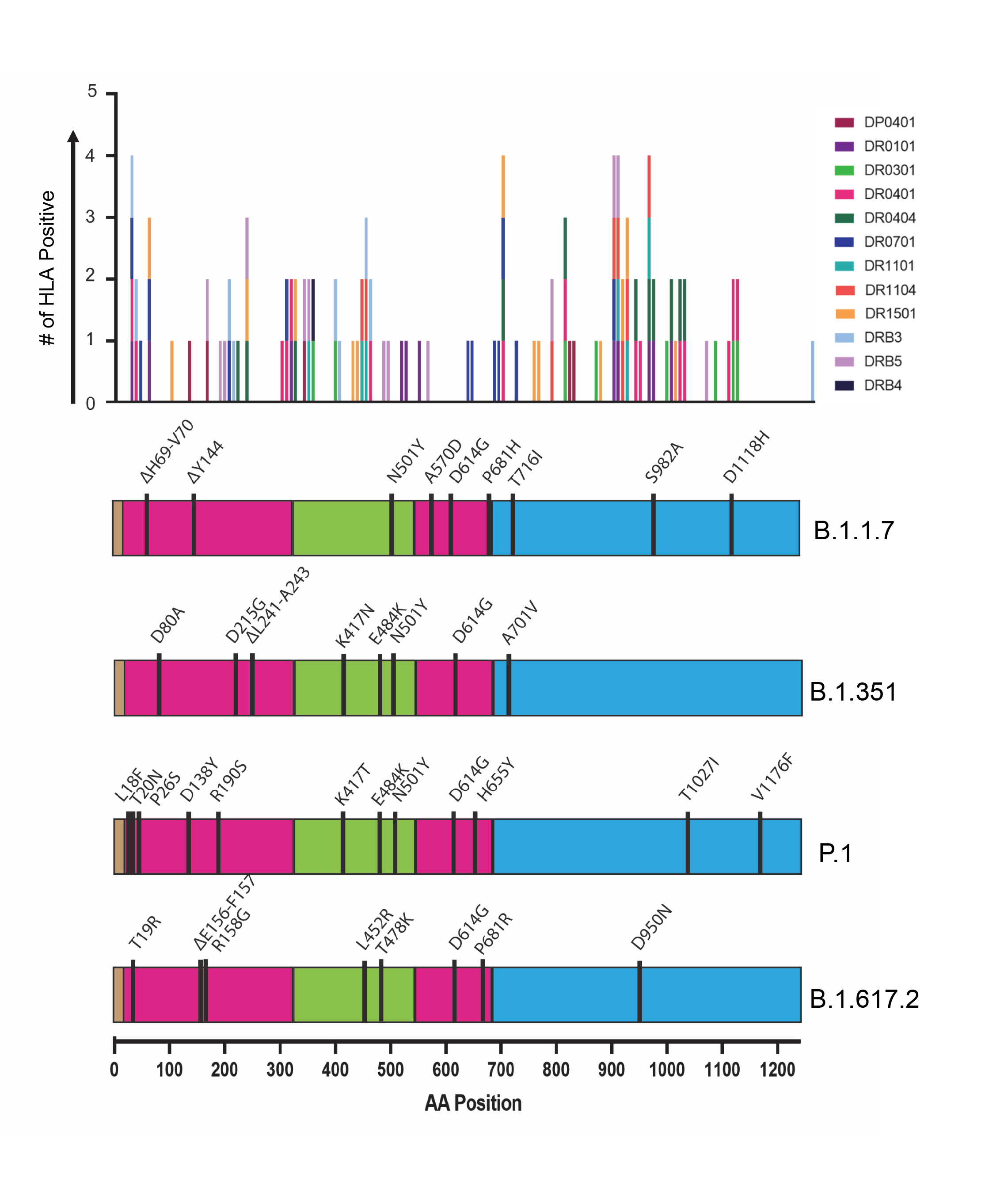

Supplement: S6 Fig — The above shows the Spike protein mutations in four prevalent SARS-CoV-2 strains, B.1.1.7, B.1.351, P.1, and B.1.617.2. Tan indicates the signal peptide, pink indicates S1, green RBD, and blue S2. (TIF) [file ppat.1010203.s006.tif]
