# Supplementary material for: Cross-reactive and mono-reactive SARS-CoV-2 CD4+ T cells in prepandemic and COVID-19 convalescent individuals
Source: PLoS Pathog. 2021 Dec 29;17(12):e1010203. doi: 10.1371/journal.ppat.1010203 (PMC8769337; doi:10.1371/journal.ppat.1010203)
Supplement: S2 Table — (DOCX) [file ppat.1010203.s008.docx]

**S2 Table. HLA-DR and DP allele frequency**
