# Supplementary material for: Cross-reactive and mono-reactive SARS-CoV-2 CD4+ T cells in prepandemic and COVID-19 convalescent individuals
Source: PLoS Pathog. 2021 Dec 29;17(12):e1010203. doi: 10.1371/journal.ppat.1010203 (PMC8769337; doi:10.1371/journal.ppat.1010203)
Supplement: S3 Table — (DOCX) [file ppat.1010203.s009.docx]

**S3 Table. Tetramer reagents used in combinatorial tetramer staining.**

| **HLA** | **Peptide** | **Epitope** | **Amino Acid Sequence** | **Streptavidin Fluorochrome** |
| --- | --- | --- | --- | --- |
| **DR0101** | Sp4 | Spike 25-44 | PPAYTNSFTRGVYYPDKVFR | PE, PE-CF594 |
|  | Sp8 | Spike 57-76 | PFFSNVTWFHAIHVSGTNGT | PE, PE-CF594 |
|  | Sp40 | Spike 313-332 | YQTSNFRVQPTESIVRFPNI | PE, PE-CF594 |
|  | Sp64 | Spike 505-524 | YQPYRVVVLSFELLHAPATV | PE, PE-Cy7 |
|  | Sp68 | Spike 537-556 | KCVNFNFNGLTGTGVLTESN | PE, PE-Cy7 |
|  | Sp112 | Spike 865-884 | GAGAALQIPFAMQMAYRFNG | PE, BV421 |
|  | Sp120 | Spike 953-972 | NQNAQALNTLVKQLSSNFGA | PE, BV421 |
|  | Sp125 | Spike 993-1012 | IDRLITGRLQSLQTYVTQQL | PE, BV421 |
|  | Sp121 | Spike 961-980 | TLVKQLSSNFGAISSVLNDI | PE, PE-CF594 |
|  | Mp11 | Membrane 71-87 | YRINWITGGIAIAMACL | PE, PE-Cy7 |
|  | Mp14 | Membrane 105-124 | RTRSMWSFNPETNILLNVPL | PE, PE-Cy7 |
|  | Np49 | NP385-404 | RQKKQQTVTLLPAADLDDFS | PE-CF594, BBV421 |
|  |  |  |  |  |
| **DR0301** | Sp44 | Spike 345-364 | TRFASVYAWNRKRISNCVAD | PE, PE-CF594 |
|  | Sp45 | Spike 353-372 | WNRKRISNCVADYSVLYNSA | PE, PE-CF594 |
|  | Sp50 | Spike 393-412 | TNVYADSFVIRGDEVRQIAP | PE, PE-CF594 |
|  | Sp101 | Spike 801-820 | NFSQILPDPSKPSKRSFIED | PE, PE-Cy7 |
|  | Sp108 | Spike 857-876 | GLTVLPPLLTDEMIAQYTSA | PE, PE-Cy7 |
|  | Sp124 | Spike 985-1004 | DKVEAEVQIDRLITGRLQSL | PE, BV421 |
|  | Sp135 | Spike 1073-1092 | KNFTTAPAICHDGKAHFPRE | PE, BV421 |
|  | Sp139 | Spike 1105-1124 | TQRNFYEPQIITTDNTFVSG | PE, BV421 |
|  | Np28 | NP 217-236 | AALALLLLDRLNQLESKMSG | PE, PE-CF594 |
|  | Np42 | NP329-348 | TWLTYTGAIKLDDKDPNFKD | PE, PE-CF594 |
|  | Mp19 | Membrane 145-164 | LRGHLRIAGHHLGRCDIKDL | PE, PE-Cy7 |
|  | Mp21 | Membrane 161-180 | IKDLPKEITVATSRTLSYYK | PE, PE-Cy7 |
|  | FluBp44 | Flu B HA 258-274 | GRIVVDYMVQKPGKTGT | PE-CF594, BV421 |
|  | TT p76 | Tetanus toxoid 1068-1077 | ITGLGAIREDNNITLKLDRC | PE-Cy7, BV421 |
|  | TT p7 | Tetanus toxoid 506-525 | NYSLDKIIVDYNLQSKITLP | PE-Cy7, BV421 |
|  |  |  |  |  |
| **DR0401** | Sp4 | Spike 25-44 | PPAYTNSFTRGVYYPDKVFR | PE, PE-CF594 |
|  | Sp5 | Spike 33-52 | TRGVYYPDKVFRSSVLHSTQ | PE, PE-CF594 |
|  | Sp38 | Spike 297-316 | SETKCTLKSFTVEKGIYQTS | PE, PE-Cy7 |
|  | Sp40 | Spike 313-332 | YQTSNFRVQPTESIVRFPNI | PE, PE-Cy7 |
|  | Sp58 | Spike 457-476 | RKSNLKPFERDISTEIYQAG | PE, BV421 |
|  | Sp101 | Spike 801-820 | NFSQILPDPSKPSKRSFIED | PE-CF594, PE-Cy7 |
|  | Sp117 | Spike 929-948 | SAIGKIQDSLSSTASALGKL | PE-CF594, BV421 |
|  | Sp127 | Spike 1009-1028 | TQQLIRAAEIRASANLAATK | PE-CF594, BV421 |
|  | Sp138 | Spike 10097-1116 | SNGTHWFVTQRNFYEPQIIT | PE-Cy7, BV421 |
|  | Sp139 | Spike 1105-1124 | TQRNFYEPQIITTDNTFVSG | PE-Cy7, BV421 |
|  | Mp13 | Membrane 97-116 | IASFRLFARTRSMWSFNPET | PE, BV421 |
|  | Mp22 | Membrane 169-188 | TVATSRTLSYYKLGASQRVA | PE, BV421 |
|  | Mp26 | Membrane 201-220 | IGNYKLNTDHSSSSDNIALL | PE, BV421 |
|  | Np7 | NP 49-68 | TASWFTALTQHGKEDLKFPR | PE-CF594, PE-Cy7 |
|  | Np16 | NP 121-140 | LPYGANKDGIIWVATEGALN | PE-CF594, PE-Cy7 |
|  | Np21 | NP 161-180 | LPQGTTLPKGFYAEGSRGGS | PE-CF594, BV421 |
|  | Np40 | NP 313-332 | AFFGMSRIGMEVTPSGTWLT | PE-CF594, BV421 |
|  | Flu MP54 | Flu MP 97-116 | VKLYRKLKREITFHGAKEIS | PE, PE-CF594 |
|  | Flu MPp16 | Flu MP 61-75 | GFVFTLTVPSERGLQ | PE, PE-CF594 |
|  | Flu HA306 | Flu HA 306-318 | PKYVKQNTLKLAT | PE, PE-Cy7 |
|  | Flu HAp68 | Flu HA 269-283 | RYAFAMERNAGSGII | PE, PE-Cy7 |
|  |  |  |  |  |
| **DR1501** | Sp8 | Spike 57-76 | PFFSNVTWFHAIHVSGTNGT | PE, PE-CF594 |
|  | Sp13 | Spike 97-116 | KSNIIRGWIFGTTLDSKTQS | PE, PE-CF594 |
|  | Sp87 | Spike 689-708 | SQSIIAYTMSLGAENSVAYS | PE, PE-Cy7 |
|  | Sp94 | Spike 745-764 | DSTECSNLLLQYGSFCTQLN | PE, PE-Cy7 |
|  | Sp126 | Spike 1001-1020 | LQSLQTYVTQQLIRAAEIRA | PE, BV421 |
|  | Sp41 | Spike 321-340 | QPTESIVRFPNITNLCPFGE | PE-Cy7, BV421 |
|  | Sp54 | Spike 425-444 | LPDDFTGCVIAWNSNNLDSK | PE-Cy7, BV421 |
|  | Sp109 | Spike 865-884 | LTDEMIAQYTSALLAGTITS | PE, BV421 |
|  | Sp115 | Spike 913-932 | QNVLYENQKLIANQFNSAIG | PE, BV421 |
|  | FluBp46 | Flu B HA 270-286 | GKTGTIVYQRGVLLPQK | PE-CF594, BV421 |
|  |  |  |  |  |
| **DRB3** | Sp4 | Spike 25-44 | PPAYTNSFTRGVYYPDKVFR | PE-CF594, PE-Cy7 |
|  | Sp5 | Spike 33-52 | TRGVYYPDKVFRSSVLHSTQ | PE-CF594, PE-Cy7 |
|  | Sp27 | Spike 209-228 | PINLVRDLPQGFSALEPLVD | Pe-CF594, BV421 |
|  | Sp50 | Spike 393-412 | TNVYADSFVIRGDEVRQIAP | Pe-CF594, BV421 |
|  | Sp57 | Spike 449-468 | YNYLYRLFRKSNLKPFERDI | PE-Cy7, BV421 |
|  | Sp58 | Spike 457-476 | RKSNLKPFERDISTEIYQAG | PE-Cy7, BV421 |
|  | Np28 | NP 217-236 | AALALLLLDRLNQLESKMSG | PE, BV421 |
|  | Sp157 | Spike 1249-1268 | SCGSCCKFDEDDSEPVLKGV | PE-CF594, PE-Cy7 |
|  |  |  |  |  |
| **DRB5** | Sp24 | Spike 185-204 | NFKNLREFVFKNIDGYFKIY | PE-CF594, PE-Cy7 |
|  | Sp30 | Spike233-252 | INITRFQTLLALHRSYLTPG | PE-CF594, PE-Cy7 |
|  | Sp43 | Spike 337-356 | PFGEVFNATRFASVYAWNRK | PE-CF594, PE-Cy7 |
|  | Sp62 | Spike 489-508 | YFPLQSYGFQPTNGVGYQPY | PE-CF594, PE-Cy7 |
|  | Sp70 | Spike 553-572 | TESNKKFLPFQQFGRDIADT | PE-CF594, PE-Cy7 |
|  | Sp98 | Spike 777-796 | NTQEVFAQVKQIYKTPPIKD | PE-CF594, BV421 |
|  | Sp112 | Spike 865-884 | GAGAALQIPFAMQMAYRFNG | PE-CF594, BV421 |
|  | Sp133 | Spike 1057-1076 | PHGVVFLHVTYVPAQEKNFT | PE-CF594, BV421 |
|  | Np7 | NP 49-68 | TASWFTALTQHGKEDLKFPR | PE, PE-CF594 |
|  | Np11 | NP 81-100 | DDQIGYYRRATRRIRGGDGK | PE, PE-CF594 |
|  | Mp22 | Membrane 169-188 | TVATSRTLSYYKLGASQRVA | PE, PE-Cy7 |
|  | Np33 | NP 257-276 | KPRQKRTATKAYNVTQAFGR | PE-CF594, PE-Cy7 |
|  | Np41 | NP 321-340 | GMEVTPSGTWLTYTGAIKLD | PE-CF594, PE-Cy7 |
|  | TT p33 | Tetanus toxoid 257-276 | QEIYMQHTYPISAEELFTFG | PE-Cy7, BV421 |
|  | TT p88 | Tetanus toxoid 1154-1173 | APSYTNGKLNIYYRRLYNGL | PE-Cy7, BV421 |
|  |  |  |  |  |
| **DP0401** | Sp17 | Spike 129-148 | KVCEFQFCNDPFLGVYYHKN | PE-CF594, PE-Cy7 |
|  | Sp21 | Spike 161-180 | SSANNCTFEYVSQPFLMDLE | PE-CF594, BV421 |
|  | Sp43 | Spike 337-356 | PFGEVFNATRFASVYAWNRK | PE-CF594, PE-Cy7 |
|  | Sp102 | Spike 809-828 | PSKPSKRSFIEDLLFNKVTL | PE-CF594, PE-Cy7 |
|  | Flu MP p47 | Flu MP 41-60 | VLMEWLKTRPILSPLTKGIL | PE-Cy7, BV421 |
