# Supplementary material for: Cross-reactive and mono-reactive SARS-CoV-2 CD4+ T cells in prepandemic and COVID-19 convalescent individuals
Source: PLoS Pathog. 2021 Dec 29;17(12):e1010203. doi: 10.1371/journal.ppat.1010203 (PMC8769337; doi:10.1371/journal.ppat.1010203)
Supplement: S4 Table — Red designated 100% AA identity to SARS-CoV-1. Green designated AA sequence with 1–3 AA mismatch to SARS CoV-1. (DOCX) [file ppat.1010203.s010.docx]

**Supplementary Table 4. Supplementary Table 4. SARS-CoV-2 Spike peptides identified in tetramer-guided epitope mapping (TGEM) with amino acid sequence identity to SARS-CoV-1. Red designated 100% AA identity to SARS-CoV-1. Green designated AA sequence with 1-3 AA mismatch to SARS CoV-1.**
